# Developmental immune network of airway lymphocytes and innate immune cells in patients with stable COPD

**DOI:** 10.3389/fimmu.2025.1614655

**Published:** 2025-06-16

**Authors:** Lanlan Liu, Mei Zhou, Shengwen Sun, Long Chen, Dehu Li, Jiaxi Lv, Rui Cheng, Jianchu Zhang, Jianghua Wu, Xianzhi Xiong

**Affiliations:** ^1^ Department of Respiratory and Critical Care Medicine, Hubei Province Clinical Research Center for Major Respiratory Diseases, Key Laboratory of Pulmonary Diseases of National Health Commission, Union Hospital, Tongji Medical College, Huazhong University of Science and Technology, Wuhan, China; ^2^ Department of Critical Care Medicine, Union Hospital, Tongji Medical College, Huazhong University of Science and Technology, Wuhan, China; ^3^ Institute of Hematology, Union Hospital, Tongji Medical College, Huazhong University of Science and Technology, Wuhan, China; ^4^ Undergraduate Research Interest Group, Department of Respiratory and Critical Care Medicine, Union Hospital, Tongji Medical College, Huazhong University of Science and Technology, Wuhan, China; ^5^ Biological Sciences Class 2302, College of Life Sciences and Technology, Huazhong Agricultural University, Wuhan, China; ^6^ Department of Medical Oncology, Sichuan Clinical Research Center for Cancer, Sichuan Cancer Hospital & Institute, Sichuan Cancer Center, University of Electronic Science and Technology of China, Chengdu, China

**Keywords:** chronic obstructive pulmonary disease, single-cell RNA sequencing, T cell exhaustion, alveolar macrophages, lipid metabolic reprogramming

## Abstract

**Introduction:**

Chronic obstructive pulmonary disease (COPD) is characterized by persistent airway inflammation and immune dysfunction. However, the molecular alterations and precise origins of immune cells in COPD airways remain poorly understood.

**Methods:**

Here, CD45+ immune cells in bronchoalveolar lavage fluid and peripheral blood mononuclear cells were collected from four COPD patients and four healthy smokers to provide a comprehensive single-cell transcriptomic atlas of immune cells in COPD airways.

**Results:**

Notably, CD8+ T cells exhibited increased exhaustion, reduced cytotoxicity, and decreased TCR diversity in COPD airways. Especially, we identified two distinct exhausted CD8+ T cell clusters (CD8Tex_PDCD1 and CD8Trm_LAG3) originating from different developmental trajectories. Regulatory T cells had a reduced proportion and regulatory capacity in COPD airways, while CD4+ tissue-resident memory T cells displayed excessive Th2 responses and diminished Th1 responses. Additionally, monocyte-derived alveolar macrophages (Macro_SPP1) underwent lipid metabolic reprogramming and exhibited a shift to an anti-inflammatory phenotype with reduced phagocytosis and protease-antiprotease imbalance in COPD airways. Furthermore, macrophages (particularly Macro_SPP1) showed increased interactions with T cells via SPP1 and GALECTIN signaling, likely contributing to T cell suppression in COPD airways.

**Conclusion:**

Together, these findings elucidate the dysregulated immune responses in COPD airways and provide a valuable resource for identifying potential therapeutic targets to restore immune homeostasis in COPD.

## 1 Introduction

Chronic obstructive pulmonary disease (COPD) is a heterogeneous lung disease characterized by persistent and progressive airflow obstruction due to abnormalities of airways (bronchitis, bronchiolitis) and alveoli (emphysema), posing a significant healthcare burden worldwide (1). The etiology of COPD is multifactorial and intricate, involving genetic susceptibility, smoking, oxidative stress, infections, and other factors (2, 3). Although smoking is the leading environmental risk factor for COPD, fewer than 50% of heavy smokers develop the disease (4). This observation underscores the individual heterogeneity and complex pathogenesis of COPD, motivating further investigation into its underlying mechanisms. Immune dysfunction is widespread in COPD, such as impaired phagocytosis and antigen presentation of macrophages, reduced antiviral responses of CD8+ T cells, and inappropriate activation and diminished pathogen recognition of mucosal-associated invariant T (MAIT) cells (5–7). These alterations contribute to chronic inflammation and compromised immune defenses.

Recent studies have mapped lung tissue and airway cells of COPD patients at single-cell resolution, revealing the alterations in the phenotypes, abundance, gene expression, and cellular interactions of structural cells, innate immune cells, and adaptive immune cells (8–11). For example, alveolar macrophages (AMs) exhibit a shift to an M2 phenotype, increased susceptibility to ferroptosis, dysregulated lipid metabolism, and mitochondrial disturbances in COPD (12–15). Additionally, CD8+KLRG1+ terminally differentiated effector memory CD45RA+ T (Temra) cells are more abundant in COPD lungs, driving pulmonary inflammation and tissue destruction (16). Moreover, T cell-derived IFN-γ may suppress the regeneration of distal airway basal cells, leading to the loss of terminal airway-enriched secretory cells and contributing to distal airway remodeling in COPD (10). Despite these advances, our knowledge of the phenotypic, functional, and interactional changes in immune cells from COPD airways remains incomplete and fragmented.

Thus, we employed single-cell RNA sequencing (scRNA-seq) and single-cell T cell receptor (TCR) sequencing (scTCR-seq) to generate a comprehensive single-cell atlas of immune cells in bronchoalveolar lavage fluid (BALF) and peripheral blood mononuclear cells (PBMC) from healthy smokers (HS) and COPD patients. This approach aims to uncover potential immunological mechanisms underlying COPD.

## 2 Methods

### 2.1 Study population and sample processing

This study was conducted in accordance with the Declaration of Helsinki and approved by the Ethics Committee of Union Hospital, Tongji Medical College, Huazhong University of Science and Technology (No. 2019/S877). Four HS and four initially diagnosed COPD patients were enrolled, and all participants provided written informed consent. COPD patients were diagnosed according to the guidelines of the global initiative for chronic obstructive lung disease (GOLD, 2018). All COPD patients were current smokers and had not received systemic treatment, including anticholinergics and glucocorticoids, within three months prior to the study. The HS cohort consisted of current smokers with a smoking history of more than 20 pack-years and normal lung function. Current smokers were defined as individuals who had smoked within the last three months prior to bronchoscopy. Participants were excluded if they had a history of malignant tumors, unstable cardiac disease, allergic and autoimmune diseases, or other acute or chronic pulmonary diseases such as asthma, bronchiectasis, fibrosis, pneumonia, or sarcoidosis. **Supplementary Table S1** summarizes the demographic and clinical characteristics of the participants.

Paired BALF and peripheral blood samples were collected from each participant. BALF was obtained from the middle lobe of the right lung of the participants via bronchoscopy at Union Hospital, Tongji Medical College, Huazhong University of Science and Technology. The BALF was diluted with phosphate buffered saline (PBS) to a final volume of 50 mL and filtered through a 100 μm nylon cell strainer (ThermoFisher Science). After centrifugation at 300 g for 10 minutes, the supernatant was removed. Then, cells were stained with CD45 antibodies, and CD45+ immune cells were isolated through flow sorting for downstream 10x scRNA-seq, scTCR-seq, and flow cytometry analyses. Peripheral blood was drawn on the same day as bronchoscopy. Peripheral blood mononuclear cells were isolated using Ficoll-Hypaque gradient centrifugation (Pharmacia, Uppsala, Sweden) and resuspended in PBS for subsequent 10x scRNA-seq, scTCR-seq, and flow cytometry analyses.

### 2.2 Single-cell RNA library preparation and sequencing

Following the manufacturer’s instructions (10x Genomics, Pleasanton, CA), Chromium Single Cell 5’ Library & Gel Bead Kit (PN-1000006) was utilized for single-cell capture and library preparation. In brief, the cell suspension, barcoded gel beads, and partitioning oil were introduced into the 10x Genomics Chromium Kit to form single-cell Gel Beads-in-Emulsion (GEMs). Captured cells were lysed, and transcripts were barcoded through reverse transcription within individual GEMs. The cDNA, along with the corresponding cell barcodes, was then amplified via PCR. Libraries for scRNA-seq were constructed using the 5’ Library Kits (PN-1000006), and libraries for scTCR-seq were prepared using the V(D)J Enrichment Kits for Human T Cells (PN-10000005). Each sample was processed independently without a hashing cell. Sequencing was performed on an Illumina NovaSeq 6000 platform.

### 2.3 scRNA-seq data processing

The 10x Chromium scRNA-seq data were processed using the CellRanger toolkit (v.6.0.0) for alignment, barcode assignment, and unique molecular identifier (UMI) counting (using the GRCh38 human reference genome). The Seurat package (v.4.1.1) (17) in R (v.4.3.1) was used to analyze the filtered gene expression matrices. Quality control (QC) was applied based on three metrics: (1) the number of detected genes was between 200 and 6,000; (2) the percentage of mitochondrial genes was below 10%; and (3) doublets were identified and removed using the R package DoubletFinder (v.2.0.3) (18). After QC filtering, a total of 62,738 single cells were retained for subsequent analysis.

### 2.4 Unsupervised clustering and marker identification

Data were normalized and scaled using the Seurat functions NormalizeData and ScaleData, and highly variable genes (HVGs) were identified using FindVariableGenes. Principal component analysis (PCA) was performed using the top 2,000 HVGs, and the top 20 principal components (PCs) were selected as the features in the PCA space. To enable joint analysis across samples, we applied the Harmony algorithm (19) for batch effect correction. The integration efficacy was validated by comparative visualization of cellular distributions before and after correction (**Supplementary Figures S1A, B**). Clustering was performed using the Seurat functions FindNeighbors and FindClusters, and dimensionality reduction was visualized using Uniform Manifold Approximation and Projection (UMAP) via the RunUMAP function (20). Cluster markers were identified using the FindAllMarkers function, and clusters were annotated using canonical and signature markers. Clusters expressing two or more sets of canonical markers of cell types were classified as doublets or undefined cells and excluded from further analysis.

To characterize cell types in detail, 1–3 rounds of clustering were performed on BALF and PBMC separately. The first round of clustering (resolution = 0.5) identified three cell types: T/NK cells, myeloid cells, and B cells. The second round of clustering was performed on T/NK cells and myeloid cells to further characterize subsets. Then, we performed the third round of clustering on CD4+ T and CD8+ T cells. The second and third rounds of clustering were performed using the top 20–30 PCs and the resolution ranging from 0.2 to 0.6.

### 2.5 Differential gene expression and pathway enrichment analysis

We identified differentially expressed genes (DEGs) based on the Wilcoxon rank-sum test using the Seurat function FindMarkers. Unless noted otherwise, we selected the genes with *P* value < 0.05 and min.pct > 0.1 as significant DEGs. Pathway enrichment analysis was performed using the enricher and GSEA functions in the clusterProfiler package (v.4.2.2) (21). Gene identifiers were mapped using the R package org.Hs.eg.db, and pathway terms were obtained from the msigdbr package (v.7.5.1), including Kyoto Encyclopedia of Genes and Genomes, Gene Ontology (GO), Hallmark, and Reactome databases. Pathways with *P* value < 0.05 were considered significantly enriched.

### 2.6 Gene set variation analysis

Gene set variation analysis (GSVA) was performed using the GSVA package (v.1.42.0) (22) to predict the functional states of CD8+ T cell and macrophage subtypes. Gene sets were obtained from the msigdbr package (v.7.5.1), and heatmaps displayed the mean expression level of each cell cluster.

### 2.7 Gene set score analysis

The AUCell package (v.1.16.0) was used to score individual cells for signature gene sets derived from previous literature (23–28). Cytotoxicity- and phagocytosis-related gene sets were obtained from the cytolysis and phagocytosis pathway terms in the GO Biological Process category. Details of the gene sets are provided in **Supplementary Table S2**.

### 2.8 Similarity analysis

Spearman’s rank correlation was used to evaluate the similarity across CD8+ T cell clusters. The Spearman’s rank correlation was calculated using the mean expression values of the top 2,000 HVGs of each cell cluster.

### 2.9 Trajectory analysis

Cell pseudotime trajectories were inferred by Monocle2 using the monocle package (v.2.26.0) (29). Marker genes for each cluster were identified using the differentialGeneTest function, and the top 2,000 genes with the lowest q-values were used for pseudotime ordering via the reduceDimension and orderCells functions. Dimensionality reduction and visualization were performed using DDRTree and plot_cell_trajectory functions. After the cell trajectories were constructed, DEGs along the pseudotime were detected using the differentialGeneTest function. Branch-dependent gene expression patterns were identified using branched expression analysis modeling (BEAM). A specialized heatmap generated by the visCluster function in the ClusterGVis package (v.0.1.1) visualized the top 2,000 branch-dependent genes. Additionally, trajectory analysis was also performed using diffusion maps via the Destiny package (v.3.8.1) (30), and connectivity between cell clusters was assessed using the partition-based graph abstraction (PAGA) algorithm in Scanpy (31).

### 2.10 TCR analysis

Raw data of scTCR-seq from 16 samples were processed using Cell Ranger (v.6.0.0) against the GRCh38 human VDJ reference genome. TCR analysis was performed using the immunarch (v.0.9.0) and scRepertoire (v.1.12.0) packages. We used alpha-beta T cells to quantify unique clonotypes scaled to the total number of clonotypes recovered based on gene sequencing. The frequency of clonotypes was categorized by the number of cell counts and classified as single, small, medium, large, and hyperexpanded clonotypes. We calculated the Shannon index to measure clonal diversity and analyzed the clonal homeostasis and clonal overlap of T cells.

### 2.11 Cell communication analysis

CellChat v2 package (32) was used to explore cell-cell communication networks via ligand-receptor interactions. The CellChatDB was set as “Secreted Signaling”, and separate cellchat objects were generated for HS and COPD groups. Interaction comparisons were performed between COPD and HS groups using the compareInteractions and RankNet functions, and the upregulated/downregulated signaling pathways were identified.

### 2.12 Flow cytometry

The expression of surface markers and intracellular molecules of cells was determined using flow cytometry. Cells were stained with fluorochrome-conjugated antibodies, which were purchased from BD Biosciences or Biolegend. Immune cells were surface-stained with fluorochrome-conjugated antibodies. The samples were incubated with antibodies for 15 min at 4°C. Cells were resuspended in PBS and washed at 400 g for 6 min. After fixation and permeabilization (eBioscience), intracellular proteins were labeled with the corresponding mAbs conjugated with fluorescent molecules, according to the manufacturer’s instructions. Flow cytometry was performed on a BD LSRFortessa X-20 and analyzed with FlowJo V10 software.

### 2.13 Statistical analysis

All statistical analyses were implemented via R (v.4.3.1) or Graphpad prism 9. For continuous variables, *t*-tests or Wilcoxon tests were used to compare differences between two groups, while Kruskal-Wallis tests were performed for multiple groups. Correlations between variables were estimated with Pearson or Spearman correlation analysis. Statistical significance was defined as *P* value < 0.05.

## 3 Results

### 3.1 Single-cell transcriptomic profiling of immune cells in the airway and peripheral blood

**Figure 1:**
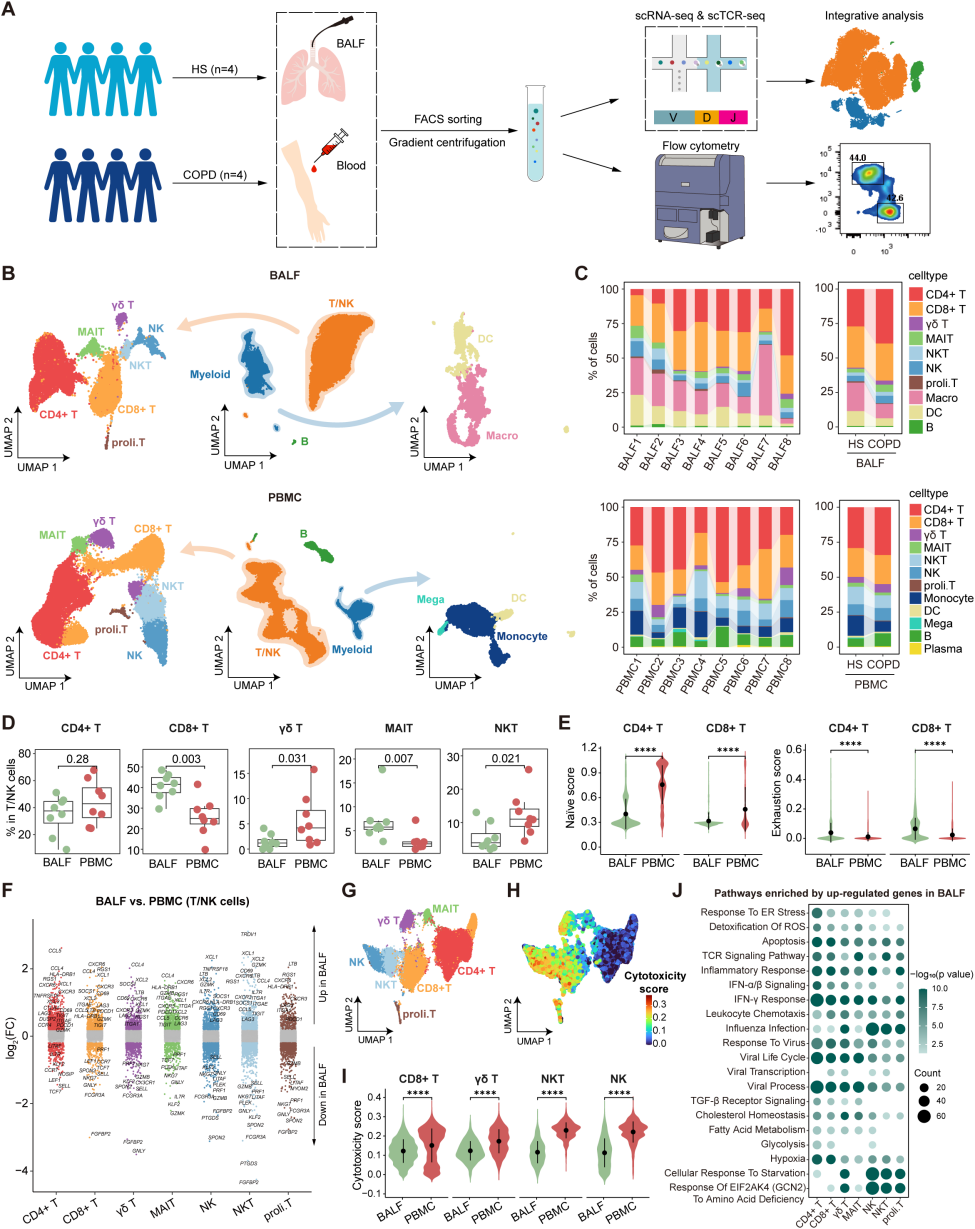
Single-cell atlas of immune cells in bronchoalveolar lavage fluid (BALF) and peripheral blood mononuclear cells (PBMC). **(A)** Schematic diagram depicting the workflow of the study design and analysis. **(B)** Combined UMAP plots showing major immune cell types in BALF (top) and in PBMC (bottom). **(C)** Major cell type proportions in BALF (top) and PBMC (bottom) samples between HS and COPD groups, colored by cell types. **(D)** Boxplots showing the proportion of T/NK cell types in BALF and PBMC. *P* value was calculated by Student’s *t* test. BALF, n = 8; PBMC, n = 8. **(E)** Violin plots showing the naïve and exhaustion scores of CD4+ T and CD8+ T cells in BALF and PBMC, calculated by AUCell. **(F)** Differentially expressed genes (DEGs) in each T/NK cell type between BALF and PBMC, performed by FindMarkers. Points with an *P* value < 0.05 and |average log_2_(fold change)| > 0.2 were colored by the cell type, otherwise they were gray. **(G)** UMAP plot showing the integrated T/NK cells from BALF and PBMC. **(H)** UMAP plot showing the cytotoxicity scores (calculated by AUCell) of integrated T/NK cells from BALF and PBMC. **(I)** Violin plots showing the cytotoxicity scores (calculated by AUCell) of CD8+ T, γδ T, NKT, and NK cells in BALF and PBMC. **(J)** Pathway enrichment analysis of T/NK cell types using the upregulated DEGs (*P* value < 0.05 and log_2_(fold change) > 0.25) in BALF compared to PBMC, performed by enricher. Dot color shows the -log_10_ (*P* value) of the pathways; dot size indicates the counts of the pathways. ****P ≤ 0.0001 by Wilcoxon test **(E, I)**.

To explore the immunological characteristics of COPD, we performed single-cell transcriptomic analysis of immune cells in BALF and PBMC from HS and COPD patients (**Figure 1A**). Following QC and filtering, we obtained raw data for 62,738 cells for downstream analysis, including 18,697 cells from BALF and 44,041 cells from PBMC (**Supplementary Table S3**). Clustering analysis was performed on BALF and PBMC samples, respectively. Firstly, initial clustering characterized three same major cellular compartments in BALF and PBMC, encompassing T/NK cells (*CD3D*, *CD4*, *CD8A*, and *NKG7*), myeloid cells (*CD14*, *CD68*, *FCER1A*, and *CD1C*), and B cells (*JCHAIN*, *MS4A1*, *CD79A*, and *CD19*) (**Figure 1B**, **Supplementary Figures S1C, D**, **Supplementary Table S4**). Then, T/NK cells showed the identical cell types between BALF and PBMC, including CD4+ T, CD8+ T, γδ T, MAIT, NKT, NK, and proliferating T (proli.T) cells (**Figure 1B**). However, myeloid cells had distinct cell types in different sample types, with macrophages and dendritic cells (DCs) in BALF but monocytes, DCs, and megakaryocytes in PBMC (**Figure 1B**), demonstrating the accuracy of our data analysis. B/plasma cells only constituted a minor fraction of BALF and PBMC (**Figure 1C**). Importantly, all cell types were derived from multiple patients (**Figure 1C**), confirming that cells were clustered according to immune characteristics rather than patient specificity.

Next, we compared the proportions and features of T/NK cell types between different anatomic compartments. Among T/NK cells, CD8+ T and MAIT cells showed higher percentages in BALF, while γδ T and NKT cells exhibited higher proportions in PBMC (**Figure 1D**). Flow cytometry data demonstrated a lower CD4/CD8 ratio in BALF than PBMC (**Supplementary Figures S2A, B**), indicating the predominance of CD8+ T cells in the airway. Further analysis of CD62L and CD45RA expression by flow cytometry revealed distinct T cell subset distributions (**Supplementary Figure S2C**). Compared to PBMC, CD4+ T cells showed reduced naive T (Tn) cell frequencies but increased effector memory T (Tem) cell proportions in BALF, and CD8+ T cells displayed lower proportions of central memory T (Tcm) and Temra cells but higher percentages of Tem cells in BALF (**Supplementary Figure S2C**). Then, we performed gene set score analysis and DEG analysis for the integrated T/NK cells from BALF and PBMC. Both CD4+ T and CD8+ T cells demonstrated lower naïve scores and reduced expression of Tn cell markers (e.g., *SELL*, *CCR7*, and *LEF1*) in BALF compared to PBMC (**Figures 1E, F**, **Supplementary Table S5**). Moreover, they upregulated the expression of tissue-resident markers (e.g., *CD69*, *ITGAE*, and *CXCR3*) in BALF, which was corroborated by flow cytometry demonstrating elevated expression of CD69, CD103, CXCR3, and CCR6 in CD4+ T and CD8+ T cells from BALF (**Figure 1F**, **Supplementary Figure S2D**). These findings highlight their identity as tissue-resident memory T cells (Trms) in the airway compartment. Importantly, CD4+ T and CD8+ T cells showed higher exhaustion signatures in BALF than PBMC, which was confirmed by elevated expression of PD-1 in BALF (**Figure 1E**, **Supplementary Figure S2E**). CD8+ T, γδ T, NKT, and NK cells were identified as the predominant cytotoxic lymphocyte populations (**Figures 1G, H**). Notably, these cells exhibited lower cytotoxicity scores and reduced expression of cytotoxic molecules (GZMB and perforin) in BALF compared to PBMC (**Figure 1I**, **Supplementary Figure S3**). Pathway enrichment analysis revealed that T/NK cells in BALF upregulated the genes associated with metabolic pathways (hypoxia, glycolysis, and fatty acid metabolism) and stress responses (cellular response to starvation, response to virus, IFN-α/β signaling, IFN-γ response, and response to endoplasmic reticulum stress) compared to their PBMC counterparts (**Figure 1J**), indicating the different immune microenvironment between the airway and peripheral blood.

In conclusion, our analysis reveals compartment-specific immune status in the airway, especially Trms with heightened exhaustion and reduced cytotoxicity, likely driven by local microenvironmental cues.

### 3.2 Dysfunctional CD8+ T cells in COPD airways

**Figure 2:**
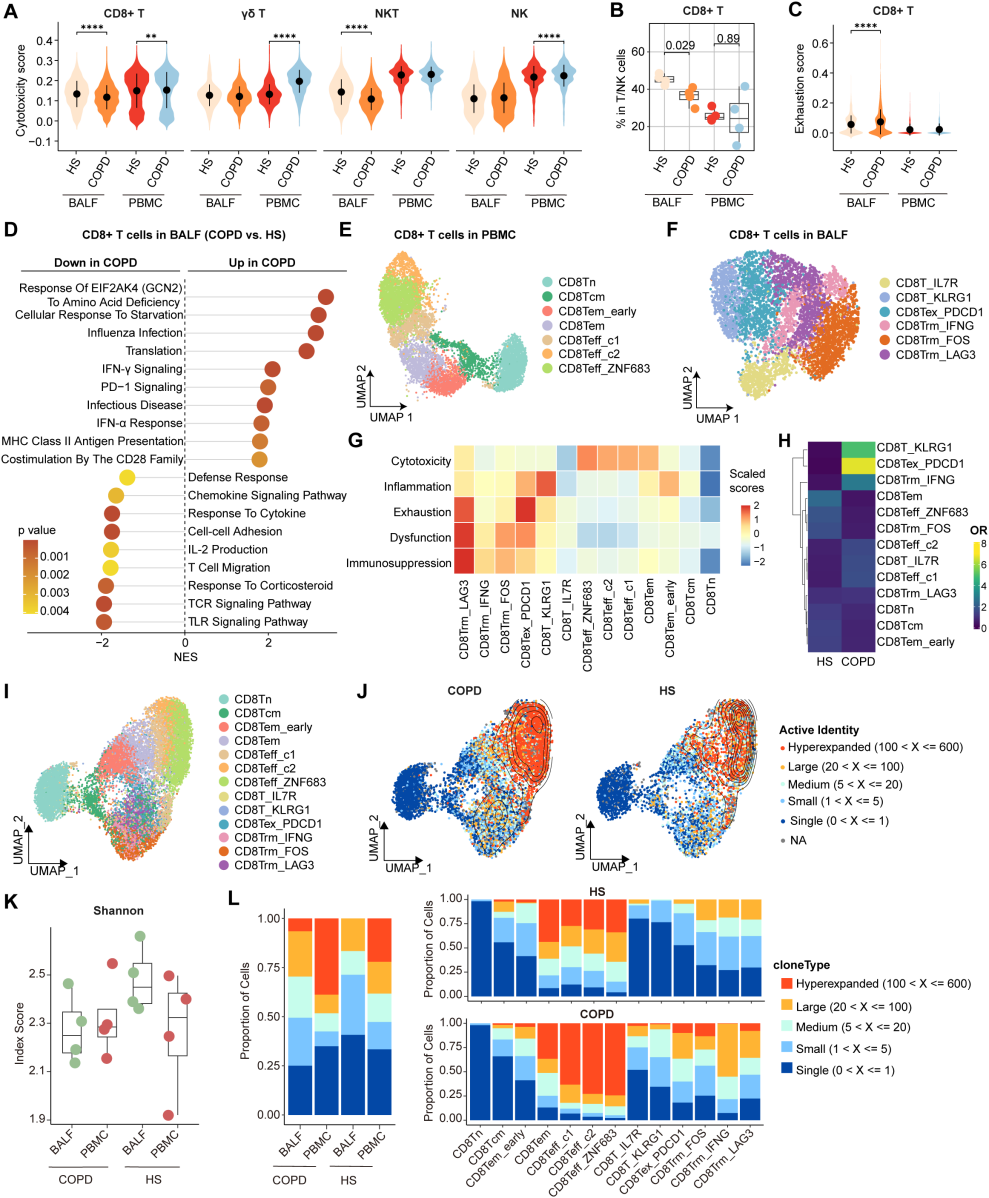
Characterization of airway CD8+ T cells in HS and COPD patients. **(A)** Violin plots showing the cytotoxicity scores of CD8+ T, γδ T, NKT, and NK cells in BALF and PBMC from HS and COPD groups, calculated by AUCell. **(B)** Boxplots showing the proportion of CD8+ T cells in BALF from HS (n = 4), BALF from COPD (n = 4), PBMC from HS (n = 4), and PBMC from COPD (n = 4). *P* value was calculated by Student’s *t* test. **(C)** Violin plots showing the exhaustion scores of CD8+ T cells in BALF and PBMC from HS and COPD groups, calculated by AUCell. **(D)** Lollipop chart showing the enriched pathways in airway CD8+ T cells from HS and COPD groups. NES, normalized enrichment score. The NES and *P* value were calculated using gene set enrichment analysis (GSEA). **(E, F)** UMAP plots showing CD8+ T cell subsets in PBMC **(E)** and in BALF **(F)**. **(G)** Heatmap showing signature gene set scores (scaled) in all CD8+ T cell subsets, calculated by AUCell. **(H)** Heatmap showing the odds ratios (ORs) of CD8+ T cell subsets occurring in HS and COPD groups. OR > 1.5 indicates that the subset is preferred to distribute in the corresponding group. Hierarchical clustering based on cosine distance is applied for rows. **(I)** UMAP plot showing integrated CD8+ T cells from BALF and PBMC. **(J)** Contour density diagram of TCR analysis showing the gradient of the expanded CD8+ T cells in COPD and HS groups. **(K)** Shannon index showing diversity of TCR repertoire of CD8+ T cells in BALF and PBMC from HS and COPD groups. **(L)** Clonal expansion status of CD8+ T cells in BALF and PBMC from HS and COPD groups (left), and clonal expansion status of all CD8+ T cell subsets from HS and COPD groups (right). **P < 0.01 and ****P < 0.0001 by Wilcoxon test **(A, C)**.

Compared with HS, PBMC CD8+ T cells showed higher cytotoxicity scores in COPD, whereas BALF CD8+ T cells exhibited lower cytotoxicity scores in COPD (**Figure 2A**). CD8+ T cells from BALF had a lower proportion and higher exhaustion scores in COPD than HS, while no significant differences were observed in PBMC (**Figures 2B, C**). Thus, we further focused on CD8+ T cells in the airway. As expected, BALF CD8+ T cells upregulated *PDCD1* expression but downregulated expression of effector function-associated genes (e.g., *GZMB*, *IFNG*, and *TNF*) in COPD compared to HS (**Supplementary Figure S4A**). Flow cytometry analysis further corroborated the elevated PD-1 expression and concomitant reduction in GZMB and perforin levels in CD8+ T cells from COPD airways (**Supplementary Figures S4B, C**). Furthermore, compared to HS, CD8+ T cells from COPD airways downregulated the functional pathways, including cell-cell adhesion, T cell migration, defense response, IL-2 production, and TCR signaling; conversely, they upregulated the T cell exhaustion-associated pathways, such as influenza infection, PD-1 signaling, IFN-γ signaling, and response of EIF2AK4 (GCN2) to amino acid deficiency (**Figure 2D**).

On deep phenotyping, CD8+ T cells in PBMC were divided into a Tn cluster (CD8Tn), a Tcm cluster (CD8Tcm), two Tem clusters (CD8Tem_early and CD8Tem), and three effector T cell (Teff) clusters (CD8Teff_c1, CD8Teff_c2, and CD8Teff_ZNF683) (**Figure 2E**, **Supplementary Figure S5A**). In BALF, CD8+ T cells were subdivided into six clusters (**Figure 2F**). Concretely, CD8T_IL7R and CD8T_KLRG1 clusters were Tcm (*IL7R*, *LEF1*, and *CCR7*) and Tem/Temra (*KLRG1*, *GZMK*, *EOMES*, *CST7*, and *S1PR5*) cells, respectively (**Supplementary Figure S5B**). CD8Tex_PDCD1 cells highly expressed exhaustion markers (*PDCD1*, *HAVCR2*, *TIGIT*, and *CTLA4*) and late activation markers (e.g., *HLA-DRA*, *HLA-DRB1*, and *HLA-DRB5*) (**Supplementary Figure S5B**), consistent with previous studies showing the co-expression of *HLA-DR* alongside the characteristic exhaustion phenotype of CD8+ T cells (33, 34). In comparison, CD8Trm_IFNG, CD8Trm_FOS, and CD8Trm_LAG3 clusters exhibited high expression of tissue-resident markers (*CD69* and *ITGAE*) and high activities of Trm differentiation-related pathways, including TGF-β signaling, IL-15 signaling, and integrin signaling (**Supplementary Figures S5B, C**). CD8Trm_IFNG cluster highly expressed cytokines and chemokines (e.g., *IFNG*, *TNF*, *XCL1*, and *XCL2*), while CD8Trm_FOS cluster showed high expression of genes associated with TCR signaling (e.g., *FOS*, *JUN*, and *NR4A1*) (**Supplementary Figure S5B**). CD8Trm_LAG3 cells were characterized by high expression of exhaustion markers (*LAG3*, *PDCD1*, *HAVCR2*, and *CTLA4*) and IFN-stimulated genes (ISGs; e.g., *IFI44*, *ISG15*, and *ISG20*) (**Supplementary Figure S5B**), reflecting a terminal exhaustion state driven by chronic IFN-I stimulation (35). By examining signature gene sets, we observed distinct functional status for each CD8+ T cell subset, with the highest cytotoxicity scores of CD8Teff, the highest inflammation scores of CD8T_KLRG1, the highest exhaustion scores of CD8Tex_PDCD1, and the highest dysfunction and immunosuppression scores of CD8Trm_LAG3 (**Figure 2G**). These findings reveal two different exhausted CD8+ T (CD8+ Tex) cell subsets (CD8Tex_PDCD1 and CD8Trm_LAG3) in the airway.

Notably, CD8T_KLRG1 and CD8Tex_PDCD1 clusters were preferentially enriched in COPD airways compared to HS (**Figure 2H**). Both CD8Tex_PDCD1 and CD8Trm_LAG3 clusters showed higher exhaustion and inflammation but lower cytotoxicity scores, while CD8Teff clusters exhibited higher cytotoxicity scores in COPD (**Supplementary Figure S5D**). We further analyzed the TCR clonotypes of all CD8+ T cell subsets from BALF and PBMC. The distinct patterns of clonal expansion indicated heterogeneous TCR repertoires between HS and COPD (**Figures 2I, J**). Compared to HS, COPD patients showed reduced TCR diversity of CD8+ T cells, as evidenced by the decreasing trend of Shannon indices in both BALF and PBMC (**Figure 2K**). Hyperexpanded TCR clonotypes accounted for 38.8% of CD8+ T cells in COPD blood compared to 22.1% in HS blood (**Figure 2L**, **Supplementary Table S6**). 6.6% of CD8+ T cells were hyperexpanded in COPD airways, whereas no hyperexpanded TCR clonotypes were detected in HS airways (**Figure 2L**, **Supplementary Table S6**). CD8Teff cells exhibited predominant hyperexpanded clonotypes, with greater clonal expansion in COPD compared to HS (**Figure 2L**, **Supplementary Table S6**). Within COPD airways, hyperexpanded TCR clonotypes were predominantly enriched in CD8Tex_PDCD1 cells (**Figure 2L**, **Supplementary Table S6**), suggesting that the hyperexpansion of a minority of clonotypes may contribute to their increased overall abundance.

Together, these data indicate increased exhaustion, impaired cytotoxicity, and reduced TCR diversity of CD8+ T cells in COPD airways.

### 3.3 Developmental trajectories of two distinct CD8+ Tex subsets in the airway

**Figure 3:**
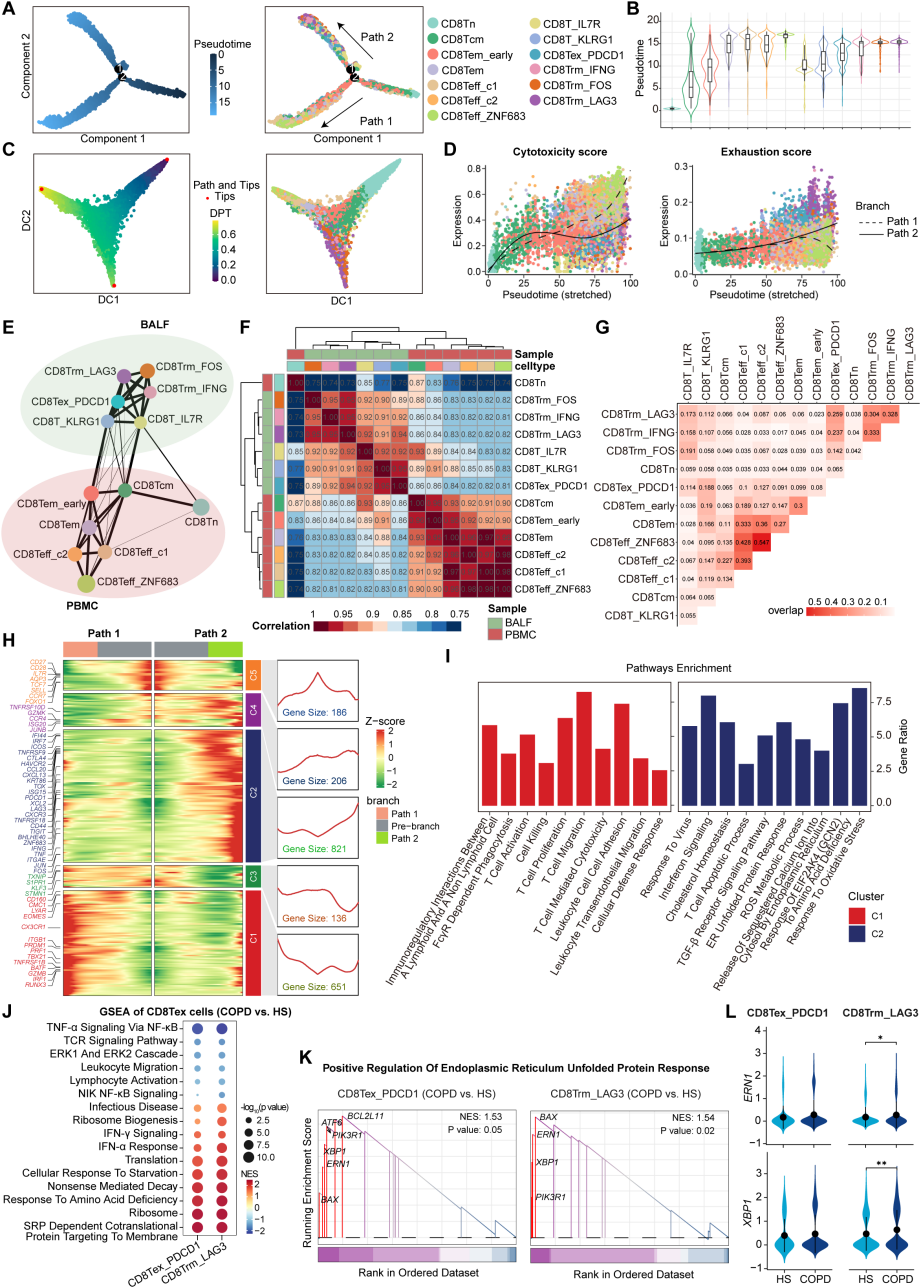
Phenotype transition of CD8+ T cells based on both TCR sharing and trajectory analysis. **(A)** The branch trajectories of CD8+ T cells inferred by Monocle2, colored by pseudotime (left) and cell types (right). **(B)** Violin plots showing the pseudotime of each CD8+ T cell subset, colored by cell types. **(C)** Diffusion maps showing the trajectories of CD8+ T cells, colored by DPT (left) and cell types (right). **(D)** Scatter distribution plots showing the cytotoxicity scores (left) and exhaustion scores (right) (calculated by AUCell) in each branch during the pseudotime of CD8+ T cells, colored by cell types. The fit curves represent the signature scores of two branches. **(E)** Partition-based graph abstraction (PAGA) analysis of all CD8+ T cell subsets. The line width (the weight of an edge) reflects a statistical measure of connectivity. **(F)** Heatmap showing the Spearman’s rank correlation between CD8+ T cell subsets. **(G)** Heatmap showing the TCR overlap of all CD8+ T cell subsets. **(H)** Heatmap (left) showing the dynamic changes along the pseudotime in expression of top 2,000 genes identified by branched expression analysis modeling (BEAM) dependent on branch point 2. The line chart (right) showing the number and the expression of the five cluster genes. **(I)** Bar plots showing the enriched pathways of Cluster 1 genes (left) and Cluster 2 genes (right) from panel **(H)**, performed by enricher. **(J)** Bubble heatmap showing the pathway activities in CD8Tex subsets from COPD and HS groups. The NES and *P* value were calculated using GSEA. Dot size reflects the -log_10_(*P* value) of pathways; dot color shows NES of pathways. **(K)** GSEA results highlighting the endoplasmic reticulum unfolded protein response related pathway activated in CD8Tex subsets from COPD patients. **(L)** Violin plots showing the expression of *ERN1* and *XBP1* in CD8Tex subsets from HS and COPD groups. *P ≤ 0.05 and **P < 0.01 by Wilcoxon test.

Next, we combined gene expression and TCR data to construct the potential developmental trajectories for all CD8+ T cells. On a global scale, CD8+ T cells could differentiate from Tn cells to either Teff (Path 1, effector trajectory) or Tex cells (Path 2, exhaustion trajectory). Monocle2 analysis showed that CD8Tn cells were at the beginning of the trajectory, whereas CD8Teff_ZNF683 cells and CD8Trm_LAG3 cells were at the terminal state of Path 1 and Path 2, respectively (**Figures 3A, B**, **Supplementary Table S7**). Such developmental trajectories for Teff and Tex cells were further supported by diffusion map analysis (**Figure 3C**). Cytotoxicity scores were gradually increased along the pseudotime in Path 1, while exhaustion scores were progressively increased in Path 2 (**Figure 3D**), further confirming the differentiation process of Teff cells and Tex cells, respectively. Next, PAGA analysis showed that CD8Tcm and CD8Tem_early clusters in PBMC exhibited high connectivity with CD8T_IL7R and CD8T_KLRG1 clusters in BALF, respectively (**Figure 3E**). Meanwhile, correlation analysis revealed the high similarity between CD8Tcm and CD8T_IL7R as well as the high similarity between CD8Tem_early and CD8T_KLRG1 (**Figure 3F**). Combined with the pseudotime data (**Figure 3B**), these results suggest that peripheral blood CD8Tcm and CD8Tem_early cells may migrate into the airway and subsequently differentiate into CD8T_IL7R and CD8T_KLRG1 cells. Then, TCR analysis showed that CD8T_KLRG1 cells exhibited a high degree of clonal overlap with CD8Tex_PDCD1 cells (**Figure 3G**). Combined with the pseudotime data, these findings suggest that CD8Tex_PDCD1 cells may develop from CD8T_KLRG1 cells; similarly, CD8T_IL7R cells may differentiate into CD8Trms and ultimately terminate in CD8Trm_LAG3 cells (**Figures 3B, G**). Hence, there are two potential exhaustion paths for airway CD8+ T cells: one via Tem cells to CD8Tex_PDCD1 cells and the other via Trms to CD8Trm_LAG3 cells.

Subsequently, we investigated the transcriptional changes associated with the trajectory branch point using BEAM analysis and observed that branch-dependent genes were categorized into five clusters (**Figure 3H**). Cluster 1 genes (e.g., *CX3CR1*, *PRF1*, and *GZMB*) showed progressive upregulation along Path 1 pseudotime, related to cytotoxic T cell differentiation, whereas Cluster 2 genes (e.g., *PDCD1*, *LAG3*, *CTLA4*, and *HAVCR2*) increased along Path 2, associated with T cell exhaustion (**Figure 3H**). In contrast to the enrichment of T cell activation, proliferation, cytotoxicity, and migration pathways in Cluster 1 genes, Cluster 2 genes were involved in response to virus, oxidative stress, amino acid deficiency, and cholesterol homeostasis (**Figure 3I**), which can trigger endoplasmic reticulum (ER) stress and unfolded protein response (UPR) (36, 37). Furthermore, both CD8Tex_PDCD1 and CD8Trm_LAG3 cells in COPD airways upregulated the pathways associated with protein synthesis and processing, such as infectious disease, ribosome biogenesis, translation, and SRP-dependent cotranslational protein targeting to membrane (**Figure 3J**). This may increase the protein-folding burden on the ER, potentially leading to ER stress. As expected, the UPR-related pathway and genes (*ERN1* and *XBP1*) were upregulated in CD8+ Tex cells from COPD compared to HS (**Figures 3K, L**).

Taken together, these results identify two distinct CD8Tex cell clusters originating from different developmental paths; ER stress may be a potential driver of CD8+ T cell exhaustion and dysfunction in COPD airways.

### 3.4 Dysregulation of regulatory T cells and CD4+ Trms in COPD airways

**Figure 4:**
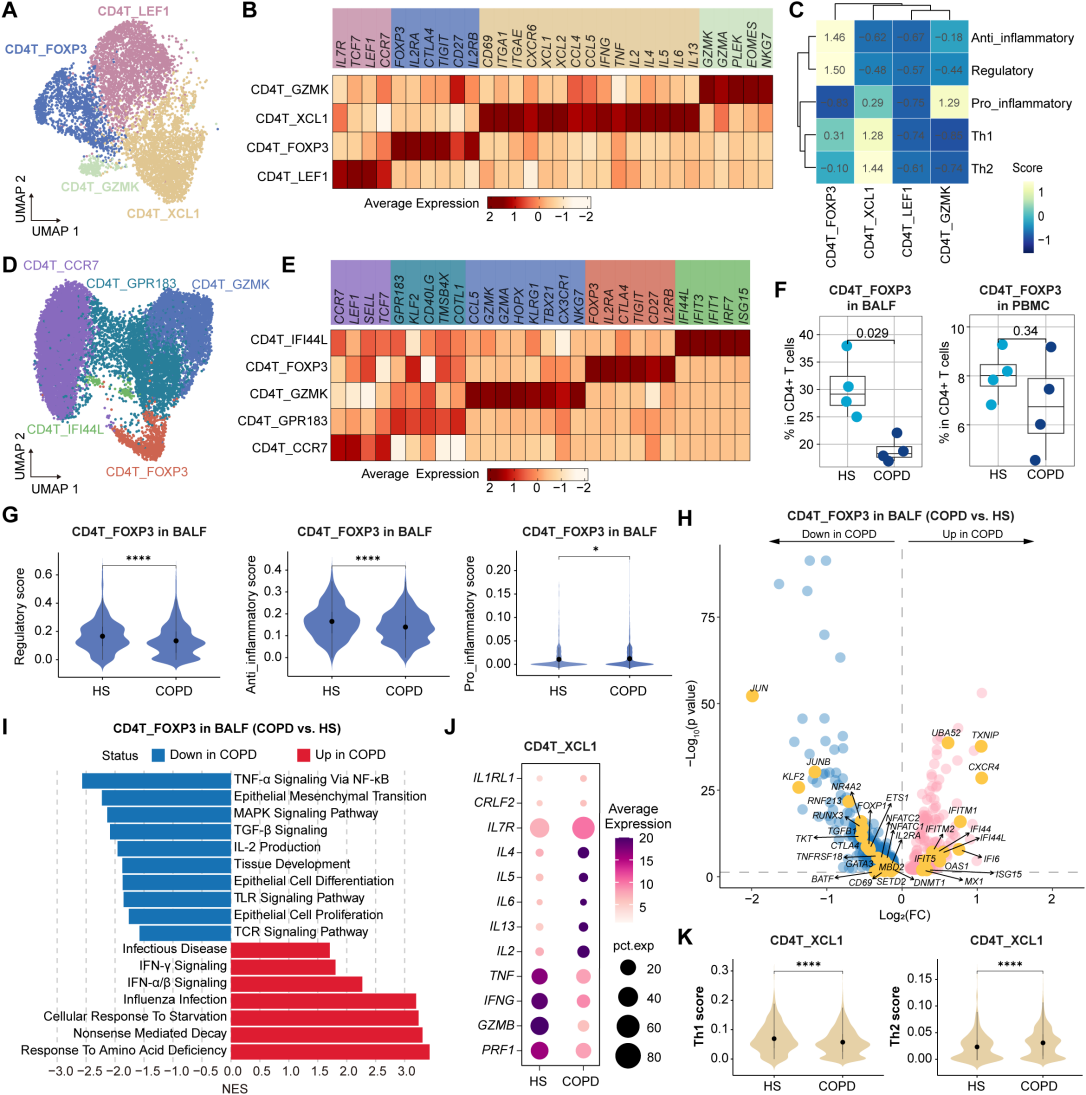
Characteristics of CD4+ T cell subsets in BALF and PBMC from HS and COPD patients. **(A)** UMAP plot showing the CD4+ T cell subsets in BALF. **(B, C)** Heatmap showing the signature genes **(B)** and gene set scores (calculated by AUCell) **(C)** in CD4+ T cell subsets from BALF. **(D)** UMAP plot showing the CD4+ T cell subsets in PBMC. **(E)** Heatmap showing the signature genes in CD4+ T cell subsets from PBMC. **(F)** Boxplots showing the proportion of CD4T_FOXP3 subset in BALF (left) and in PBMC (right) from HS and COPD groups. *P* value was calculated by Student’s *t* test. HS, n = 4; COPD, n = 4. **(G)** Violin plots showing the regulatory, anti-inflammatory, and pro-inflammatory scores of CD4T_FOXP3 subset in BALF from HS and COPD groups, calculated by AUCell. **(H)** Volcano plot showing DEGs of CD4T_FOXP3 subset in BALF between COPD and HS groups, performed by FindMarkers. **(I)** GSEA results showing the enriched pathways in CD4T_FOXP3 subset in BALF between COPD and HS groups. **(J)** Bubble heatmap showing the expression level of the function-related genes in CD4T_XCL1 subset from BALF of COPD and HS groups. Dot color indicates the average expression; dot size indicates the proportion of cells expressing the gene. **(K)** Violin plots showing the Th1 and Th2 scores in CD4T_XCL1 subset from BALF of HS and COPD groups, calculated by AUCell. *P < 0.05 and ****P < 0.0001 by Wilcoxon test **(G, K**).

We identified four clusters of CD4+ T cells in BALF (**Figure 4A**). CD4T_LEF1 and CD4T_GZMK clusters were associated with central memory (*IL7R*, *TCF7*, *CCR7*, and *LEF1*) and effector memory (*GZMK*, *GZMA*, *EOMES*, and *NKG7*), respectively (**Figure 4B**). CD4T_FOXP3 cluster exhibited high expression of *FOXP3*, *IL2RA*, *CTLA4*, and *TIGIT*, known markers of regulatory T cells (Tregs), and displayed significant anti-inflammatory and regulatory signatures (**Figures 4B, C**). CD4T_XCL1 cluster represented CD4+ Trms, strongly expressing *CD69*, *ITGAE*, and *CXCR6* (**Figure 4B**). In PBMC, CD4+ T cells were classified into a Tn cluster (CD4T_CCR7), a memory T (Tm) cluster (CD4T_GPR183), a Temra cluster (CD4T_GZMK), a Treg cluster (CD4T_FOXP3), and an ISG+ T cluster (CD4T_IFI44L) (**Figures 4D, E**).

Compared to HS, Tregs (CD4T_FOXP3 cells) exhibited a reduced proportion in COPD airways, with only a decreasing trend in COPD peripheral blood (**Figure 4F**). Next, we focused on the airway Tregs. They showed lower regulatory and anti-inflammatory scores but higher pro-inflammatory scores in COPD than in HS (**Figure 4G**). Besides, they displayed elevated expression of IFN-responsive genes (e.g., *IFI44L*, *IFI6*, *IFIT5*, *MX1*, *ISG15*, and *OAS1*) but decreased expression of several genes critical for Treg development, survival, and function, such as *JUN*, *JUNB*, *CTLA4*, *CD69*, *NR4A2*, *KLF2*, *IL2RA*, and *TGFB1* (**Figure 4H**). Additionally, airway Tregs upregulated influenza infection, IFN-α/β signaling, and IFN-γ signaling pathways, but downregulated IL-2 production, TGF-β, TNF-α, Toll-like receptor (TLR), TCR, and MAPK signaling pathways in COPD (**Figure 4I**).

CD4+ Trms are a heterogeneous population, including T-helper (Th) 1 Trms, Th2 Trms, and T follicular helper (Tfh)-like resident helper cells (38). In this study, the CD4+ Trm (CD4T_XCL1) cluster was polyfunctional, co-expressing cytotoxic molecules (*GZMA*), chemokines (*CCL4*, *CCL5*, *XCL1*, and *XCL2*), and Th1/Th2-related cytokines (*IL2*, *IFNG*, *TNF*, *IL4*, and *IL13*) (**Figure 4B**). Compared to HS, CD4+ Trms exhibited elevated Th2 signatures and genes (*IL4*, *IL5*, and *IL13*) in COPD (**Figures 4J, K**). Polyfunctional CD4+ Trms are also indispensable in protecting the lungs against respiratory pathogens. For instance, they constitutively express high transcript levels of cytotoxic mediators, such as IFN-γ (39). However, CD4+ Trms showed lower Th1 signatures and expression of *IFNG*, *TNF*, *GZMB*, and *PRF1* in COPD than HS (**Figures 4J, K**).

Collectively, these results reveal the impaired anti-inflammatory function of Tregs and imbalanced Th1/Th2 response of CD4+ Trms in COPD airways.

### 3.5 Lipid metabolic reprogramming and anti-inflammatory phenotype of macrophages in COPD airways

**Figure 5:**
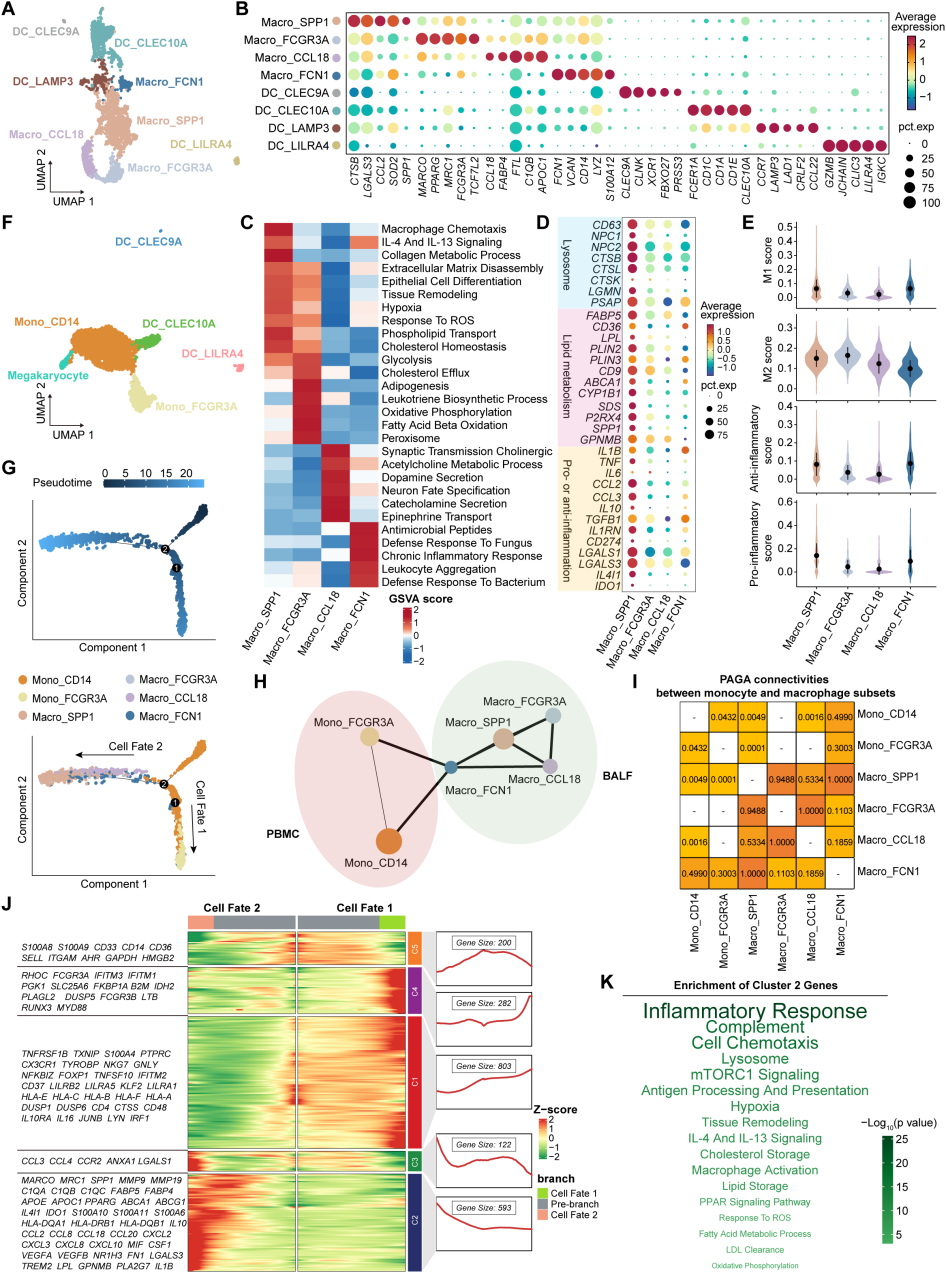
Characteristics and transition states of airway macrophages and blood monocytes. **(A)** UMAP plot showing the myeloid cell subsets in BALF. **(B)** Bubble heatmap showing the signature genes in myeloid cell subsets in BALF. Dot color indicates the average expression (scaled); dot size indicates the proportion of cells expressing the gene. **(C)** Heatmap showing the different pathway activities scored by gene set variation analysis in macrophage subsets. **(D)** Bubble heatmap showing the signature genes in macrophage subsets. Dot color indicates the average expression (scaled); dot size indicates the proportion of cells expressing the gene. **(E)** Violin plots showing the signature gene set scores of macrophage subsets, calculated by AUCell. **(F)** UMAP plot showing the myeloid cell subsets in PBMC. **(G)** The branch trajectories of airway macrophages and blood monocytes inferred by Monocle2, colored by pseudotime (top) and cell types (bottom). **(H)** PAGA analysis of airway macrophages and blood monocytes. The line width (the weight of an edge) reflects a statistical measure of connectivity. **(I)** The table summarizing the results of the PAGA connectivity calculation. A value of 1 indicates a strong connection and 0 indicates a weak connection between two cell types. **(J)** Heatmap (left) showing the dynamic changes along the pseudotime in expression of top 2,000 genes identified by BEAM dependent on branch point 2. The line chart (right) showing the number and the expression of the five cluster genes. **(K)** The enriched pathways of Cluster 2 genes from panel **(J)**, performed by enricher. The size and the color of the font indicate the -log_10_(*P* value) of pathways.

Myeloid cells were divided into four macrophage clusters and four DC clusters in BALF (**Figure 5A**). Concretely, Macro_FCGR3A cells strongly expressed *MARCO*, *MRC1*, and *PPARG* (**Figure 5B**), representing tissue-resident AMs (TRAMs) (40). They exhibited high activities of fatty acid beta oxidation, oxidative phosphorylation, and glycolysis pathways (**Figure 5C**), which were essential for maintaining long-lived, self-replenishing tissue-resident macrophages (41, 42). As the predominant macrophage subset, Macro_SPP1 cluster highly expressed lysosomal genes (e.g., *CD63*, *NPC2*, *CTSB*, and *LGMN*) and lipid metabolism-related genes (e.g., *FABP5*, *SPP1*, *GPNMB*, *ABCA1*, and *CD9*), showing the high activities of phospholipid transport, cholesterol homeostasis, collagen metabolism, and extracellular matrix (ECM) disassembly pathways (**Supplementary Figure S6A**, **Figures 5C, D**). Macro_SPP1 cluster deviated from the classical M1/M2 paradigm, as it simultaneously expressed both pro- and anti-inflammatory genes (e.g., *TNF*, *CCL2*, *IL10*, *IL1RN*, and *LGALS3*) and concurrently exhibited high pro- and anti-inflammatory scores (**Figures 5D, E**). Interestingly, SPP1+ macrophages have been described in numerous diseases, such as fatty liver (43), COVID-19 (44), dystrophic muscle (45), and cancers (46), indicating a conserved functional phenotype across diseases. Macro_FCN1 cluster highly expressed *CD14*, *VCAN*, *FCN1*, and *S100A12*, indicating monocyte-like macrophages with high responses to chronic inflammation and pathogens (**Figures 5B, C**). Macro_CCL18 cluster, marked by *CCL18*, *FABP4*, and *C1QB*, was implicated in acetylcholine metabolism and catecholamine secretion pathways (**Figures 5B, C**). In addition to the three traditional types, cDC1 (DC_CLEC9A), cDC2 (DC_CLEC10A), and pDC (DC_LILRA4), we also identified a non-classical DC type (DC_LAMP3) in BALF, characterized by high expression of *CCR7* and *LAMP3* (**Figures 5A, B**). In PBMC, we identified a classical monocyte cluster (Mono_CD14), a non-classical monocyte cluster (Mono_FCGR3A), a megakaryocyte cluster, and three traditional DC clusters mentioned above (**Figure 5F**, **Supplementary Figure S6B**).

Next, we integrated all macrophages and monocytes to investigate their developmental trajectories (**Supplementary Figure S6C**). Firstly, Monocle2 analysis revealed two distinct trajectory branches, both starting from Mono_CD14 cells and terminating at Mono_FCGR3A cells (Cell Fate 1) and Macro_SPP1 cells (Cell Fate 2), respectively (**Figure 5G**, **Supplementary Figure S6D**; **Supplementary Table S8**). Diffusion map analysis corroborated these trajectory patterns (**Supplementary Figure S6E**). Furthermore, we assessed the likelihood of connections among all subsets using the connectivity matrix of the PAGA network. Macro_SPP1 cells showed the strongest PAGA connectivity with Macro_FCN1 cells, and Macro_FCN1 cells exhibited higher connectivity with Mono_CD14 than Mono_FCGR3A cells (**Figures 5H, I**). These findings suggest that Macro_SPP1 cells are monocyte-derived AMs (MoAMs) originating from classical monocytes. Subsequently, the branch-dependent genes identified by BEAM analysis were divided into five clusters (**Figure 5J**). Notably, Cluster 2 genes (e.g., *MARCO*, *SPP1*, *C1QC*, *TREM2*, *GPNMB*, *FABP5*, *APOE*, *IL1B*, *IL10*, *CXCL8*, and *CCL2*) showed a gradual increase during the development of Macro_SPP1 cells (**Figure 5J**). Pathway enrichment analysis revealed that Cluster 2 genes were associated with hypoxia, response to reactive oxygen species (ROS), low-density lipoprotein (LDL) clearance, lysosome, fatty acid metabolism, and lipid storage pathways (**Figure 5K**). This aligns with a previous study showing that increased ROS levels promote the formation of lipid-laden macrophages in the lungs (47).

**Figure 6:**
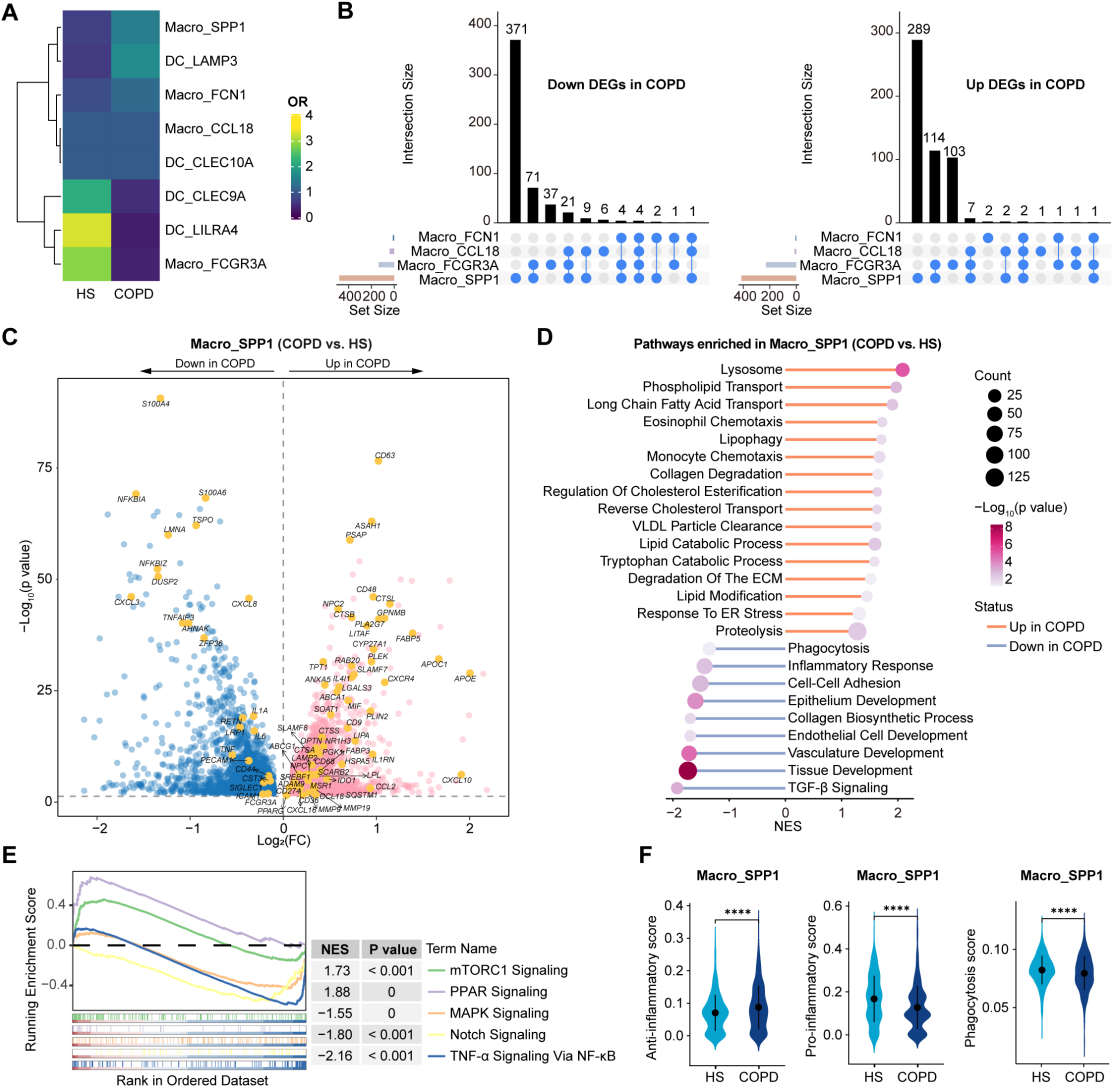
Dissection of Macro_SPP1 cells showing the altered lipid metabolism and functions in COPD airways. **(A)** Heatmap showing the ORs of airway myeloid cell subsets occurring in HS and COPD groups. OR > 1.5 indicates that the subset is preferred to distribute in the corresponding groups. Hierarchical clustering based on cosine distance is applied for rows. **(B)** Upset plots showing the overlapping and non-overlapping DEGs in macrophage subtypes between COPD and HS groups. The horizontal bar chart on the left represents the number of elements in each set, the colored points in the middle and the lines between the points represent the intersection of different macrophage subsets, and the vertical bar chart at the top represents the number of corresponding intersection elements. Left panel: downregulated DEGs in COPD; Right panel: upregulated DEGs in COPD. **(C)** Volcano plot showing DEGs in Macro_SPP1 subset between COPD and HS groups, performed by FindMarkers. **(D)** Lollipop chart showing the enriched pathways in Macro_SPP1 subset from COPD and HS groups. *P* value was calculated using GSEA. **(E)** GSEA results showing the enriched pathways in Macro_SPP1 subset from COPD. **(F)** Violin plots showing the signature gene set scores of Macro_SPP1 cells in HS and COPD groups, calculated by AUCell. ****P ≤ 0.0001 by Wilcoxon test.

Interestingly, Macro_SPP1 cells were preferentially enriched in COPD, while Macro_FCGR3A cells were in HS (**Figure 6A**). Among all macrophage subsets, Macro_SPP1 cells exhibited the largest numbers of DEGs between COPD and HS (**Figure 6B**, **Supplementary Table S9**), highlighting their high plasticity and immunoreactivity. Importantly, Macro_SPP1 cells in COPD airways might undergo lipid metabolic reprogramming. They upregulated genes involved in cholesterol and lipid transport and metabolism (e.g., *PPARG*, *CD36*, *FABP5*, *LIPA*, *SREBF1*, *SOAT1*, *ABCA1*, *APOC1*, *NR1H3*, and *APOE*) and pathways of long-chain fatty acid transport, VLDL particle clearance, lysosome, reverse cholesterol transport, and regulation of cholesterol esterification (**Figures 6C, D**). Moreover, the mTORC1 and PPAR signaling pathways, which modulated lipid metabolism and promoted anti-inflammatory phenotypes, were upregulated, while pro-inflammatory pathways such as Notch, NF-κB, and MAPK signaling were downregulated in COPD (**Figure 6E**). A recent study has demonstrated the induction of the monocyte-derived macrophage population (marked by *Spp1*, *Gpnmb*, *Fabp5*, *Cd9*, and *Arg1*) in lungs by Notch2 blockade (48). Functionally, Macro_SPP1 cells in COPD exhibited higher anti-inflammatory scores but lower phagocytosis scores, with elevated expression of immunosuppressive genes (e.g., *IL1RN*, *CCL18*, *CD274*, *IL4I1*, and *IDO1*) but decreased expression of efferocytosis-related genes (e.g., *LRP1*, *PECAM1*, *CD44*, *SIGLEC1*, *ICAM1*, and *FCGR3A*) (**Figures 6C, F**). Additionally, Macro_SPP1 cells upregulated proteolysis, collagen degradation, and degradation of the ECM pathways in COPD (**Figure 6D**). Consistently, they showed increased expression of protease genes (e.g., *ADAM9*, *CTSB*, *CTSL*, *CTSS*, and *MMP9*) but decreased expression of the antiprotease gene (*CST3*) in COPD (**Figure 6C**).

In summary, these results reveal that Macro_SPP1 cells undergo lipid metabolic reprogramming, exhibiting an anti-inflammatory phenotype, reduced phagocytosis, and protease-antiprotease imbalance in COPD airways.

### 3.6 Altered intercellular communication networks of airway immune cells in COPD

**Figure 7:**
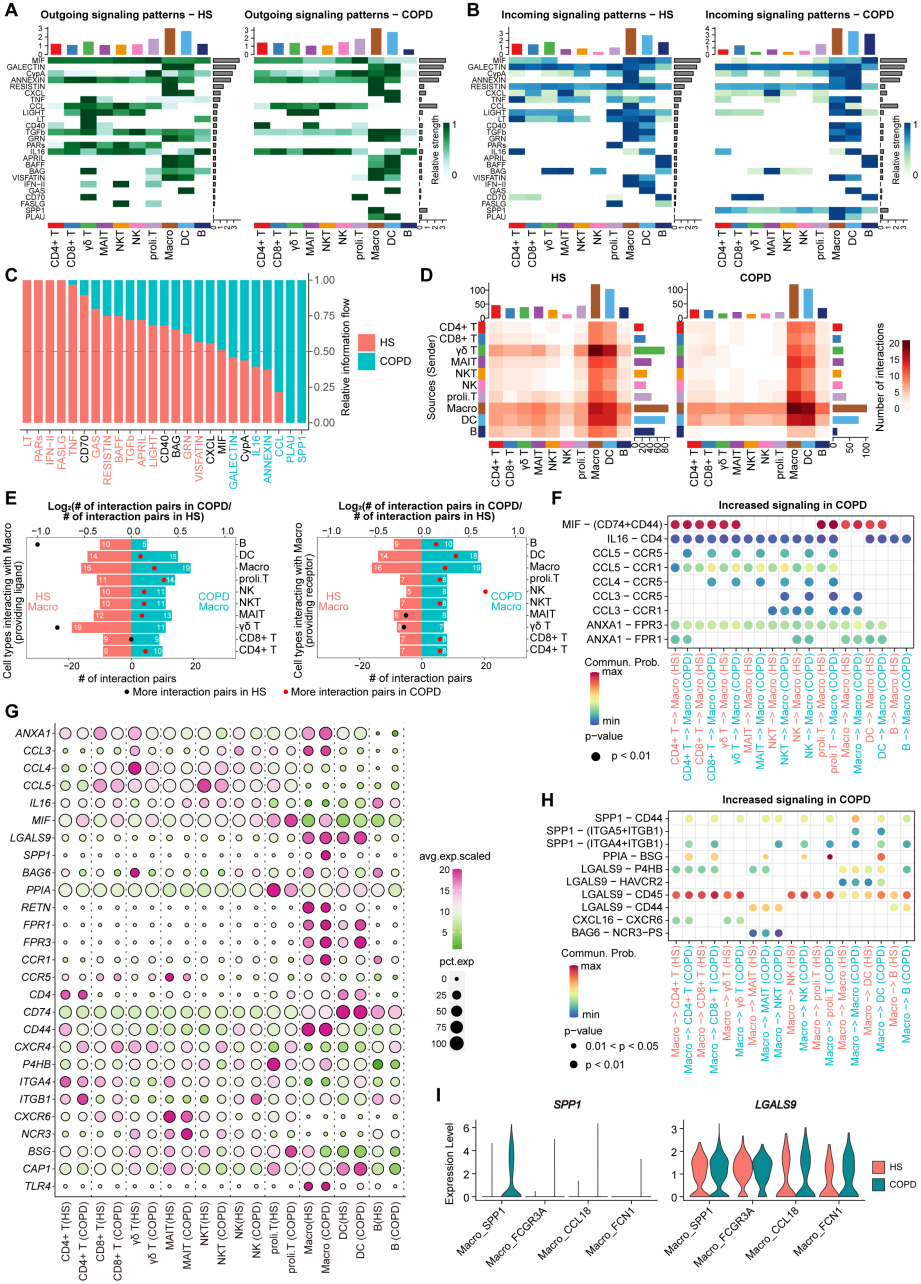
Cellular communication analysis in immune cells from HS and COPD airways. **(A)** Heatmap plots showing the outgoing communication patterns of major immune cell types both in HS (left) and COPD (right) groups. The relative strength of each signal pathway is color coded form gray to green. **(B)** Heatmap plots showing the incoming communication patterns of major immune cell types both in HS (left) and COPD (right) groups. The relative strength of each signal pathway is color coded form gray to blue. **(C)** All significant signaling pathways are ranked based on their differences in overall information flow within the inferred networks between HS and COPD groups. The left signaling pathways colored red are more enriched in HS group, the middle ones colored black are equally enriched in both groups, and the right ones colored green are more enriched in COPD group. **(D)** Heatmap showing the number of interactions between major immune cell types in HS (left) and COPD (right) groups. **(E)** Bar chart showing the number of significant ligand-receptor (L-R) pairs in macrophages and other cells in COPD and HS groups. Macrophages providing receptors (left) and ligands (right) were calculated separately. The dots represent the ratio of the number of significant L-R pairs between COPD and HS groups, the ratio above 1 in red and below 1 in black. **(F)** Bubble plot showing the increased L-R pairs from all major cell types to macrophages in COPD patients. Commun. Prob = Communication probability. The dot color and size represent the calculated communication probability and *P* values. **(G)** Bubble heatmap showing the gene expression of ligands and receptors in each major immune cell type from HS and COPD groups. Dot color indicates the average expression; dot size indicates the proportion of cells expressing the gene. **(H)** Bubble plot showing the increased L-R pairs from macrophages to all major cell types in COPD patients. Commun. Prob = Communication probability. The dot color and size represent the calculated communication probability and *P* values. **(I)** Violin plots showing the ligand gene expression of SPP1 and GALECTIN signaling in macrophage subsets between COPD and HS groups.

We performed CellChat analysis to explore potential intercellular communication patterns among airway immune cells (**Figures 7A, B**). Overall, we detected 25 significant secreted signaling pathways mediating interactions across all immune cell types in the airway (**Figure 7C**). Among these, six pathways (SPP1, PLAU, CCL, ANNEXIN, IL16, and GALECTIN) were upregulated in COPD, while 13 pathways (LT, PARs, IFN-II, FASLG, TNF, GAS, RESISTIN, BAFF, TGFb, APRIL, LIGHT, GRN, and VISFATIN) were upregulated in HS (**Figure 7C**). Although the majority of ligand-receptor (L-R) pairs mediating cell-cell interactions were shared between COPD and HS, distinct L-R pairs were also identified in each group, such as SPP1-CD44, SPP1-(ITGA5+ITGB1), and SPP1-(ITGA4+ITGB1) in COPD while LTA-TNFRSF1B, LTA-TNFRSF1A, LTA-TNFRSF14, and LTA-(LTB+LTBR) in HS (**Supplementary Figure S7**).

Notably, macrophages harbored the largest numbers of ligands and receptors, actively interacting with all cell types in both HS and COPD (**Figure 7D**). In total, macrophages in COPD received and sent more signals than HS (**Figure 7E**). Among the signals received by macrophages, we observed that MIF-(CD74+CD44), CCL3-CCR1, CCL3-CCR5, CCL4-CCR5, CCL5-CCR1, CCL5-CCR5, ANXA1-FPR1, and ANXA1-FPR3 were upregulated in COPD compared with HS (**Figure 7F**). These L-R pairs were involved in MIF, CCL, and ANNEXIN signaling pathways. The transcriptomic analysis further revealed that macrophages, CD4+ T, CD8+ T, γδ T, NK, and proli.T cells exhibited higher expression of the ligand gene *MIF*, and macrophages showed elevated expression of receptor genes *CCR1*, *CCR5*, *FPR1*, and *FPR3* in COPD compared to HS (**Figure 7G**). More importantly, among the signals sent by macrophages, SPP1-CD44, SPP1-(ITGA4+ITGB1), LGALS9-P4HB, LGALS9-CD45, and LGALS9-CD44 were upregulated in COPD compared to HS (**Figure 7H**). These L-R pairs were involved in immunosuppressive SPP1 and GALECTIN signaling pathways, by which macrophages interacted with T cells. Notably, the upregulation of these L-R pairs might be attributed to the significantly increased expression of ligand genes (*SPP1* and *LGALS9*) in macrophages from COPD (**Figure 7G**). Especially, *SPP1* and *LGALS9* were mainly upregulated in the Macro_SPP1 subset from COPD compared with HS (**Figure 7I**).

Thus, these findings suggest macrophages (particularly Macro_SPP1 cells) as key regulators of T cell dysfunction and identify SPP1 and GALECTIN signaling pathways as potential therapeutic targets for restoring immune homeostasis in COPD airways.

## 4 Discussion

In this study, we generated a comprehensive single-cell transcriptomic atlas of immune cells in the airway and peripheral blood of HS and COPD patients. Importantly, CD8+ T cells in COPD airways exhibited increased exhaustion, reduced cytotoxicity, and decreased TCR diversity. Tregs showed a reduced proportion and impaired regulatory function in COPD airways, accompanied by excessive Th2 responses and diminished Th1 responses in CD4+ Trms. Macro_SPP1 cells underwent lipid metabolic reprogramming and exhibited an anti-inflammatory phenotype in COPD airways. Furthermore, macrophages (particularly Macro_SPP1) likely suppressed T cells via SPP1 and GALECTIN signaling in COPD airways. Our findings revealed profound alterations in immune cell composition, function, and interaction in COPD airways.

Our data revealed the distinct distribution and function of T/NK cells between the airway and peripheral blood. In particular, CD8+ T cells showed higher relative abundance and tissue-resident signatures (*CD69*, *ITGAE*, and *CXCR3*) in the airway. Previous studies have also reported that CD8+ T cells are dominant in the airway and express high levels of CD69, CXCR3, CCR5, and CCR6 (49–51). These chemokine receptors (especially CXCR3) could mediate CD8+ T cell trafficking into the airways (52, 53). The unique microenvironmental cues in the airway, such as hypoxia, nutrient deprivation, viral infections, IFN stimulation, and oxidative stress, may promote T cell tissue residency and exhaustion (54, 55). Consistent with previous studies (56), we observed that airway CD8+ T cells overexpressed several exhaustion markers and inhibitory receptors (e.g., *PDCD1*, *TIGIT*, and *CTLA4*) and exhibited lower cytotoxicity. It suggests that CD8+ T cells are recruited into the airway and subsequently modulated into an exhausted and low-cytotoxicity phenotype under the influence of local environmental factors. These compartmental discrepancies may underlie the compartment-specific functional changes of T/NK cells in COPD. Particularly, we observed the increased cytotoxicity in peripheral blood CD8+ T, γδ T, and NK cells from COPD patients. Cytotoxic T and NK cells have been implicated in COPD pathogenesis, leading to emphysema and airway remodeling (57–59). Our previous study similarly reported elevated GZMB and perforin expression in peripheral blood CD4+ T, CD8+ T, γδ T, and NK cells from COVID-19 patients with pulmonary sequelae (60), suggesting that increased cytotoxicity in peripheral blood T and NK cells may correlate with pulmonary damage. Conversely, airway CD8+ T and NKT cells exhibited decreased cytotoxicity in COPD. This aligns with the impaired protective functions of CD8+ T and MAIT cells against pathogen infections in COPD (5–7). Collectively, our work highlights compartment-specific immune responses in COPD, underscoring the necessity of investigating airway immune cell characteristics to fully understand the pathogenesis.

In the airway, CD8+ T cells were more exhausted in COPD. A recent study has shown an elevated proportion of PD-1+CD8+ T cells in COPD lungs, which fail to effectively upregulate cytotoxic degranulation in response to influenza infections (6). This may explain the increased susceptibility to viral infections associated with acute exacerbations of COPD (AECOPD). Interestingly, we identified distinct developmental trajectories for the two CD8+ Tex subsets (CD8Tex_PDCD1 and CD8Trm_LAG3), reminiscent of the exhaustion paths observed in tumor-infiltrating T cells (61). Multiple pathways involved in T cell exhaustion were upregulated in COPD, including viral infection, PD-1 signaling, IFN signaling, and amino acid deficiency pathways. ER stress has been reported to promote COPD by driving mucus hypersecretion from bronchial epithelial cells, lung epithelial cell apoptosis, and smooth muscle cell autophagy (62). Interestingly, we found that ER stress and UPR might be involved in the exhaustion and dysfunction of CD8+ T cells in COPD airways. The ER stress sensor XBP1 can bind to the *Pdcd1* promoter and activate *Pdcd1* gene transcription (63). Overexpressing XBP1 increases the expression of immune checkpoints on CD8+ T cells, inducing functional exhaustion (63). Furthermore, high cholesterol-induced ER stress can disrupt endoplasmic reticulum-mitochondria contact site function in CD8+ T cells, leading to mitophagy and abnormal mitochondrial energy metabolism, ultimately inducing CD8+ T cell exhaustion (64). Therefore, targeting ER stress and UPR pathways may contribute to restoring CD8+ T cell functions in COPD.

Tregs are essential in preventing deleterious inflammation and constraining tissue damage (65). Hou et al. reported a decrease in immunosuppressive Tregs and an accumulation of pro-inflammatory Tregs with increasing emphysema severity (66). In this study, Tregs exhibited reduced regulatory and anti-inflammatory capacity in COPD airways. Interestingly, they upregulated the expression of IFN-responsive genes. These are reminiscent of the Tregs that exhibit diminished suppressive capacity following viral infections, leading to increased production of Th2-type cytokines (67). Previous studies have reported that Treg reduction and dysfunction can lead to excessive Th2 inflammation (68, 69). Our findings indicate excessive Th2 responses of CD4+ Trms in COPD airways. High Th2 signatures have been closely linked to disease severity and reduced lung function in COPD patients (70, 71). Type 2 cytokines (e.g., IL-4 and IL-13) promote mucus hypersecretion, airway remodeling, and emphysema by enhancing mucin synthesis, airway mucosal permeability, fibrin deposition, and protease production (72–77). Additionally, these cytokines can impair virus-induced IFN production by inhibiting TLR signaling in airway epithelial cells, leading to increased viral replication (78). This suggests that Th2 inflammation may heighten susceptibility to viral infections, a key trigger of AECOPD. Interestingly, polyfunctional CD4+ Trms also mediate protective immunity against respiratory pathogens (38). IFN-γ-producing CD4+ T cells are essential for the optimal formation of lung CD8+ Trms, which mediate protective responses during influenza infection (79). Independent of their helper function, CD4+ Trms directly protect against the influenza virus by producing IFN-γ and cytotoxic molecules (80, 81). Thus, the reduced Th1 responses and expression of *IFNG*, *TNF*, *GZMB*, and *PRF1* in CD4+ Trms may indicate the impaired anti-infection capacity in COPD airways. Therefore, restoring the function of Tregs and CD4+ Trms may contribute to alleviating Th2 inflammation and reducing infection susceptibility in COPD patients.

AMs can be replenished by long-lived TRAMs or monocyte-derived cells (41). In this study, we identified an SPP1+ macrophage population (Macro_SPP1) derived from classical monocytes (Mono_CD14). SPP1+ macrophages have been widely described in cancer, aging, and chronic inflammatory diseases, exhibiting conserved functional features including fibrosis promotion, extracellular matrix remodeling, and immune modulation (82). A recent study has reported that SPP1+ macrophages are at the terminal phase of the differentiation path (83), while other research suggests that these macrophages may be in an intermediate developmental state (84). Interestingly, Macro_SPP1 cells (MoAMs) appeared to replace Macro_FCGR3A cells (TRAMs) as the main source to replenish the AM pool in COPD airways. However, their reduced phagocytosis and efferocytosis in COPD airways may suggest impaired clearance of pathogens, apoptotic cells, and cellular debris, contributing to increased exacerbations and chronic inflammation in COPD (85, 86). Moreover, Macro_SPP1 cells showed elevated expression of protease genes (e.g., *ADAM9*, *CTSL*, *CTSS*, and *MMP9*) in COPD, which were associated with emphysema and airway remodeling (87–90). More importantly, they upregulated immunosuppressive factors (e.g., *CCL18*, *IL1RN*, *CD274*, *IL4I1*, and *IDO1*) in COPD airways, which can inhibit T cell proliferation and effector function (91–94). Notably, Macro_SPP1 cells upregulated the lipid metabolism-related genes (e.g., *PPARG*, *CD36*, *FABP5*, *LIPA*, *SREBF1*, and *SOAT1*) in COPD airways, suggesting that they may undergo lipid metabolic reprogramming to drive their phenotypic and functional alterations. Previous studies have shown that long-chain fatty acid transport mediated by fatty acid-binding protein 5 (encoded by *FABP5*) induces the immunosuppression of lipid-loaded macrophages through activating PPAR-γ (encoded by *PPARG*) (46, 95). Additionally, PPAR-γ activation upregulates the expression of CD36, enhancing the uptake of oxidized LDL (96). Lysosomal acid lipase (encoded by *LIPA*) promotes the lipolysis of LDL-delivered triacylglycerols, providing fatty acids for fatty acid oxidation, which is important for macrophage M2 polarization (97, 98). Moreover, sterol regulatory element binding protein 1 (encoded by *SREBF1*) promotes alternative activation of macrophages by inducing *de novo* lipogenesis and depleting antioxidant defenses (99). A recent study has reported elevated cholesteryl esters and increased lipid storage in AMs from COPD patients (100). Interestingly, Macro_SPP1 cells from COPD exhibited elevated expression of *SOAT1*, which encoded acetyl-CoA acetyltransferase 1 to convert fatty acids and free cholesterol into cholesteryl esters for storage in lipid droplets. Furthermore, lipid droplets can polarize infiltrating monocytes into M2-like macrophages by regulating the catabolism of free fatty acids for mitochondrial respiration (101). In summary, lipid metabolic reprogramming may drive the phenotypic and functional alterations of Macro_SPP1 cells, contributing to immune dysfunction and tissue damage in COPD.

Our interaction analysis further identified macrophages as key contributors to the suppression of T cell functions in COPD airways. Several chemokines (e.g., CCL3-CCR5/CCR1 and CCL5-CCR5) have been reported to recruit monocytes/macrophages and promote their M2 polarization (102). The HIF1A-FOSL2-ANXA1-FPR1/3 axis and MIF signaling network (MIF-CD74/CXCR4 and MIF-CD74/CD44) are also involved in monocyte recruitment and macrophage M2 polarization, resulting in the inhibited killing capacity of CD8+ T cells (103–106). Targeting MIF-CD74 or ANXA1 in macrophages can repolarize M2 macrophages into an M1 phenotype, reduce immunosuppressive factor expression, and relieve CD8+ T cell suppression (107–109). More importantly, macrophages, particularly the Macro_SPP1 subset, sent more SPP1 and GALECTIN signaling to T cells in COPD airways. Especially, SPP1-CD44 and LGALS9-CD45 axes are widely recognized in the interaction between tumor-associated macrophages and T cells, which suppress T cell activation and induce T cell exhaustion (110, 111). Targeting SPP1 or LGALS9 can relieve the exhausting phenotype of T cells (112–114). Therefore, our findings highlight promising targets for restoring T cell functions and remodeling the immune microenvironment in COPD airways.

In summary, we comprehensively characterized the airway and peripheral blood immune cells, although this was a small, single-center study involving 16 samples, revealing the development of lymphocytes and innate immune cells and their immune networks in COPD. Our findings have important implications for gaining a deeper understanding of COPD immunopathogenesis and may offer valuable targets and insights for addressing the immune dysfunction in COPD. We anticipate that our study will inspire further research into revealing the potential mechanisms underlying COPD subphenotypes.

## Data Availability

The datasets presented in this study will be deposited in the Sequence Read Archive (SRA) database under accession number PRJNA1236421. The core code used for data analysis is available at https://github.com/llliu-lab/COPD_scRNA_analysis.

## References

[1] Agustí A Celli BR Criner GJ Halpin D Anzueto A Barnes P. Global initiative for chronic obstructive lung disease 2023 report: gold executive summary. Am J Respir Crit Care Med. (2023) 207:819–37. doi: 10.1164/rccm.202301-0106PP PMC10111975 36856433

[2] Rabe KF Watz H. Chronic obstructive pulmonary disease. Lancet (London England). (2017) 389:1931–40. doi: 10.1016/s0140-6736(17)31222-9 28513453

[3] Christenson SA Smith BM Bafadhel M Putcha N. Chronic obstructive pulmonary disease. Lancet (London England). (2022) 399:2227–42. doi: 10.1016/s0140-6736(22)00470-6 35533707

[4] Løkke A Lange P Scharling H Fabricius P Vestbo J. Developing copd: A 25 year follow up study of the general population. Thorax. (2006) 61:935–9. doi: 10.1136/thx.2006.062802 PMC2121175 17071833

[5] Huber ME Larson E Lust TN Heisler CM Harriff MJ. Chronic obstructive pulmonary disease and cigarette smoke lead to dysregulated mucosal-associated invariant T-cell activation. Am J Respir Cell Mol Biol. (2023) 68:90–102. doi: 10.1165/rcmb.2022-0131OC 36174211 PMC9817907

[6] McKendry RT Spalluto CM Burke H Nicholas B Cellura D Al-Shamkhani A. Dysregulation of antiviral function of Cd8(+) T cells in the chronic obstructive pulmonary disease lung. Role of the Pd-1-Pd-L1 axis. Am J Respir Crit Care Med. (2016) 193:642–51. doi: 10.1164/rccm.201504-0782OC PMC4824936 26517304

[7] Chen J Wang X Schmalen A Haines S Wolff M Ma H. Antiviral Cd8(+) T cell immune responses are impaired by cigarette smoke and in copd. Eur Respir J. (2023) 62:2201374. doi: 10.1183/13993003.01374-2022 37385655 PMC10397470

[8] Sauler M McDonough JE Adams TS Kothapalli N Barnthaler T Werder RB. Characterization of the copd alveolar niche using single-cell RNA sequencing. Nat Commun. (2022) 13:494. doi: 10.1038/s41467-022-28062-9 35078977 PMC8789871

[9] Huang Q Wang Y Zhang L Qian W Shen S Wang J. Single-cell transcriptomics highlights immunological dysregulations of monocytes in the pathobiology of copd. Respir Res. (2022) 23:367. doi: 10.1186/s12931-022-02293-2 36539833 PMC9764587

[10] Rustam S Hu Y Mahjour SB Rendeiro AF Ravichandran H Urso A. A unique cellular organization of human distal airways and its disarray in chronic obstructive pulmonary disease. Am J Respir Crit Care Med. (2023) 207:1171–82. doi: 10.1164/rccm.202207-1384OC PMC10161760 36796082

[11] Booth S Hsieh A Mostaco-Guidolin L Koo HK Wu K Aminazadeh F. A single-cell atlas of small airway disease in chronic obstructive pulmonary disease: A cross-sectional study. Am J Respir Crit Care Med. (2023) 208:472–86. doi: 10.1164/rccm.202303-0534OC 37406359

[12] Baßler K Fujii W Kapellos TS Dudkin E Reusch N Horne A. Alveolar macrophages in early stage copd show functional deviations with properties of impaired immune activation. Front Immunol. (2022) 13:917232. doi: 10.3389/fimmu.2022.917232 35979364 PMC9377018

[13] Liégeois M Bai Q Fievez L Pirottin D Legrand C Guiot J. Airway macrophages encompass transcriptionally and functionally distinct subsets altered by smoking. Am J Respir Cell Mol Biol. (2022) 67:241–52. doi: 10.1165/rcmb.2021-0563OC PMC9348561 35522264

[14] Li Y Yang Y Guo T Weng C Yang Y Wang Z. Heme oxygenase-1 determines the cell fate of ferroptotic death of alveolar macrophages in copd. Front Immunol. (2023) 14:1162087. doi: 10.3389/fimmu.2023.1162087 37215140 PMC10196003

[15] Fan P Zhang Y Ding S Du Z Zhou C Du X. Integrating RNA-Seq and Scrna-Seq to explore the mechanism of macrophage ferroptosis associated with copd. Front Pharmacol. (2023) 14:1139137. doi: 10.3389/fphar.2023.1139137 36969832 PMC10036582

[16] Villaseñor-Altamirano AB Jain D Jeong Y Menon JA Kamiya M Haider H. Activation of Cd8+ T cells in copd lung. Am J Respir Crit Care Med. (2023) 208:1177–95. doi: 10.1164/rccm.202305-0924OC PMC10868372 37756440

[17] Butler A Hoffman P Smibert P Papalexi E Satija R. Integrating single-cell transcriptomic data across different conditions, technologies, and species. Nat Biotechnol. (2018) 36:411–20. doi: 10.1038/nbt.4096 PMC6700744 29608179

[18] McGinnis CS Murrow LM Gartner ZJ. Doubletfinder: doublet detection in single-cell RNA sequencing data using artificial nearest neighbors. Cell Syst. (2019) 8:329–37.e4. doi: 10.1016/j.cels.2019.03.003 30954475 PMC6853612

[19] Korsunsky I Millard N Fan J Slowikowski K Zhang F Wei K. Fast, sensitive and accurate integration of single-cell data with harmony. Nat Methods. (2019) 16:1289–96. doi: 10.1038/s41592-019-0619-0 PMC6884693 31740819

[20] Becht E McInnes L Healy J Dutertre CA Kwok IWH Ng LG. Dimensionality reduction for visualizing single-cell data using Umap. Nat Biotechnol. (2018) 37:38–44. doi: 10.1038/nbt.4314 30531897

[21] Yu G Wang LG Han Y He QY. Clusterprofiler: an R package for comparing biological themes among gene clusters. Omics: J Integr Biol. (2012) 16:284–7. doi: 10.1089/omi.2011.0118 PMC3339379 22455463

[22] Hänzelmann S Castelo R Guinney J. Gsva: gene set variation analysis for microarray and RNA-seq data. BMC Bioinf. (2013) 14:7. doi: 10.1186/1471-2105-14-7 PMC3618321 23323831

[23] Zhang C Li J Cheng Y Meng F Song JW Fan X. Single-cell RNA sequencing reveals intrahepatic and peripheral immune characteristics related to disease phases in hbv-infected patients. Gut. (2023) 72:153–67. doi: 10.1136/gutjnl-2021-325915 PMC9763233 35361683

[24] Zemmour D Zilionis R Kiner E Klein AM Mathis D Benoist C. Single-cell gene expression reveals a landscape of regulatory T cell phenotypes shaped by the Tcr. Nat Immunol. (2018) 19:291–301. doi: 10.1038/s41590-018-0051-0 29434354 PMC6069633

[25] Azizi E Carr AJ Plitas G Cornish AE Konopacki C Prabhakaran S. Single-cell map of diverse immune phenotypes in the breast tumor microenvironment. Cell. (2018) 174:1293–308.e36. doi: 10.1016/j.cell.2018.05.060 29961579 PMC6348010

[26] Yan ZX Dong Y Qiao N Zhang YL Wu W Zhu Y. Cholesterol efflux from C1qb-expressing macrophages is associated with resistance to chimeric antigen receptor T cell therapy in primary refractory diffuse large B cell lymphoma. Nat Commun. (2024) 15:5183. doi: 10.1038/s41467-024-49495-4 38890370 PMC11189439

[27] Guo R Kong J Tang P Wang S Sang L Liu L. Unbiased single-cell sequencing of hematopoietic and immune cells from aplastic anemia reveals the contributors of hematopoiesis failure and dysfunctional immune regulation. Adv Sci (Weinheim Baden-Wurttemberg Germany). (2024) 11:e2304539. doi: 10.1002/advs.202304539 PMC10933602 38145351

[28] Aizarani N Saviano A Sagar Mailly L Durand S Herman JS. A human liver cell atlas reveals heterogeneity and epithelial progenitors. Nature. (2019) 572:199–204. doi: 10.1038/s41586-019-1373-2 31292543 PMC6687507

[29] Qiu X Mao Q Tang Y Wang L Chawla R Pliner HA. Reversed graph embedding resolves complex single-cell trajectories. Nat Methods. (2017) 14:979–82. doi: 10.1038/nmeth.4402 PMC5764547 28825705

[30] Angerer P Haghverdi L Büttner M Theis FJ Marr C Buettner F. Destiny: diffusion maps for large-scale single-cell data in R. Bioinf (Oxford England). (2016) 32:1241–3. doi: 10.1093/bioinformatics/btv715 26668002

[31] Wolf FA Hamey FK Plass M Solana J Dahlin JS Göttgens B. Paga: graph abstraction reconciles clustering with trajectory inference through a topology preserving map of single cells. Genome Biol. (2019) 20:59. doi: 10.1186/s13059-019-1663-x 30890159 PMC6425583

[32] Jin S Plikus MV Nie Q. Cellchat for systematic analysis of cell-cell communication from single-cell transcriptomics. Nat Protoc. (2025) 20:180–219. doi: 10.1038/s41596-024-01045-4 39289562

[33] Mouillaux J Allam C Gossez M Uberti T Delwarde B Hayman J. Tcr activation mimics Cd127(Low)Pd-1(High) phenotype and functional alterations of T lymphocytes from septic shock patients. Crit Care (London England). (2019) 23:131. doi: 10.1186/s13054-018-2305-5 PMC6472012 30995946

[34] Wang Z Zhu L Nguyen THO Wan Y Sant S Quiñones-Parra SM. Clonally diverse Cd38(+)Hla-Dr(+)Cd8(+) T cells persist during fatal H7n9 disease. Nat Commun. (2018) 9:824. doi: 10.1038/s41467-018-03243-7 29483513 PMC5827521

[35] Chen W Teo JMN Yau SW Wong MY Lok CN Che CM. Chronic type I interferon signaling promotes lipid-peroxidation-driven terminal Cd8(+) T cell exhaustion and curtails anti-pd-1 efficacy. Cell Rep. (2022) 41:111647. doi: 10.1016/j.celrep.2022.111647 36384131

[36] Macauslane KL Pegg CL Short KR Schulz BL. Modulation of endoplasmic reticulum stress response pathways by respiratory viruses. Crit Rev Microbiol. (2024) 50:750–68. doi: 10.1080/1040841x.2023.2274840 37934111

[37] Nair KA 2nd Liu B. Navigating the landscape of the unfolded protein response in Cd8(+) T cells. Front Immunol. (2024) 15:1427859. doi: 10.3389/fimmu.2024.1427859 39026685 PMC11254671

[38] Oja AE van Lier RAW Hombrink P. Two sides of the same coin: protective versus pathogenic Cd4(+) resident memory T cells. Sci Immunol. (2022) 7:eabf9393. doi: 10.1126/sciimmunol.abf9393 35394815

[39] Oja AE Piet B Helbig C Stark R van der Zwan D Blaauwgeers H. Trigger-happy resident memory Cd4(+) T cells inhabit the human lungs. Mucosal Immunol. (2018) 11:654–67. doi: 10.1038/mi.2017.94 29139478

[40] Reyfman PA Walter JM Joshi N Anekalla KR McQuattie-Pimentel AC Chiu S. Single-cell transcriptomic analysis of human lung provides insights into the pathobiology of pulmonary fibrosis. Am J Respir Crit Care Med. (2019) 199:1517–36. doi: 10.1164/rccm.201712-2410OC PMC6580683 30554520

[41] Kulikauskaite J Wack A. Teaching old dogs new tricks? The plasticity of lung alveolar macrophage subsets. Trends Immunol. (2020) 41:864–77. doi: 10.1016/j.it.2020.08.008 PMC7472979 32896485

[42] Remmerie A Scott CL. Macrophages and lipid metabolism. Cell Immunol. (2018) 330:27–42. doi: 10.1016/j.cellimm.2018.01.020 29429624 PMC6108423

[43] Remmerie A Martens L Thoné T Castoldi A Seurinck R Pavie B. Osteopontin expression identifies a subset of recruited macrophages distinct from Kupffer cells in the fatty liver. Immunity. (2020) 53:641–57.e14. doi: 10.1016/j.immuni.2020.08.004 32888418 PMC7501731

[44] Wendisch D Dietrich O Mari T von Stillfried S Ibarra IL Mittermaier M. Sars-Cov-2 infection triggers profibrotic macrophage responses and lung fibrosis. Cell. (2021) 184:6243–61.e27. doi: 10.1016/j.cell.2021.11.033 34914922 PMC8626230

[45] Vitaliti A Reggio A Colletti M Galardi A Palma A. Integration of single-cell datasets depicts profiles of macrophages and fibro/adipogenic progenitors in dystrophic muscle. Exp Cell Res. (2024) 442:114197. doi: 10.1016/j.yexcr.2024.114197 39111382

[46] Zhang S Peng W Wang H Xiang X Ye L Wei X. C1q(+) tumor-associated macrophages contribute to immunosuppression through fatty acid metabolic reprogramming in Malignant pleural effusion. J Immunother Cancer. (2023) 11:e007441. doi: 10.1136/jitc-2023-007441 37604643 PMC10445384

[47] Zhu Y Dutta S Han Y Choi D Polverino F Owen CA. Oxidative stress promotes lipid-laden macrophage formation via Cyp1b1. Redox Biol. (2024) 79:103481. doi: 10.1016/j.redox.2024.103481 39721495 PMC11732233

[48] Cruz Tleugabulova M Melo SP Wong A Arlantico A Liu M Webster JD. Induction of a distinct macrophage population and protection from lung injury and fibrosis by Notch2 blockade. Nat Commun. (2024) 15:9575. doi: 10.1038/s41467-024-53700-9 39505846 PMC11541919

[49] Saris A Reijnders TDY Nossent EJ Schuurman AR Verhoeff J Asten SV. Distinct cellular immune profiles in the airways and blood of critically ill patients with Covid-19. Thorax. (2021) 76:1010–9. doi: 10.1136/thoraxjnl-2020-216256 PMC8050882 33846275

[50] Bryant N Muehling LM Wavell K Teague WG Woodfolk JA. Rhinovirus as a driver of airway T cell dynamics in children with treatment-refractory recurrent wheeze. JCI Insight. (2025) 10:e189480. doi: 10.1172/jci.insight.189480 40337866 PMC12128989

[51] Connors TJ Ravindranath TM Bickham KL Gordon CL Zhang F Levin B. Airway Cd8(+) T cells are associated with lung injury during infant viral respiratory tract infection. Am J Respir Cell Mol Biol. (2016) 54:822–30. doi: 10.1165/rcmb.2015-0297OC PMC4942220 26618559

[52] Li J Jing Q Hu Z Wang X Hu Y Zhang J. Mycobacterium tuberculosis-specific memory T cells in bronchoalveolar lavage of patients with pulmonary tuberculosis. Cytokine. (2023) 171:156374. doi: 10.1016/j.cyto.2023.156374 37782984

[53] Slütter B Pewe LL Kaech SM Harty JT. Lung airway-surveilling Cxcr3(Hi) memory Cd8(+) T cells are critical for protection against influenza a virus. Immunity. (2013) 39:939–48. doi: 10.1016/j.immuni.2013.09.013 PMC3872058 24238342

[54] Zheng MZM Wakim LM. Tissue resident memory T cells in the respiratory tract. Mucosal Immunol. (2022) 15:379–88. doi: 10.1038/s41385-021-00461-z PMC8526531 34671115

[55] Baessler A Vignali DAA. T cell exhaustion. Annu Rev Immunol. (2024) 42:179–206. doi: 10.1146/annurev-immunol-090222-110914 38166256

[56] Cheon IS Son YM Sun J. Tissue-resident memory T cells and lung immunopathology. Immunol Rev. (2023) 316:63–83. doi: 10.1111/imr.13201 37014096 PMC10524334

[57] Finch DK Stolberg VR Ferguson J Alikaj H Kady MR Richmond BW. Lung dendritic cells drive natural killer cytotoxicity in chronic obstructive pulmonary disease via Il-15rα. Am J Respir Crit Care Med. (2018) 198:1140–50. doi: 10.1164/rccm.201712-2513OC PMC6221577 29676596

[58] Williams M Todd I Fairclough LC. The role of Cd8 + T lymphocytes in chronic obstructive pulmonary disease: A systematic review. Inflammation Res: Off J Eur Histamine Res Soc. (2021) 70:11–8. doi: 10.1007/s00011-020-01408-z PMC7806561 33037881

[59] de Fays C Geudens V Gyselinck I Kerckhof P Vermaut A Goos T. Mucosal immune alterations at the early onset of tissue destruction in chronic obstructive pulmonary disease. Front Immunol. (2023) 14:1275845. doi: 10.3389/fimmu.2023.1275845 37915582 PMC10616299

[60] Wu J Tang L Ma Y Li Y Zhang D Li Q. Immunological profiling of Covid-19 patients with pulmonary sequelae. mBio. (2021) 12:e0159921. doi: 10.1128/mBio.01599-21 34488453 PMC8546863

[61] Zheng L Qin S Si W Wang A Xing B Gao R. Pan-cancer single-cell landscape of tumor-infiltrating T cells. Science. (2021) 374:abe6474. doi: 10.1126/science.abe6474 34914499

[62] Peng H Zhou Q Liu J Wang Y Mu K Zhang L. Endoplasmic reticulum stress: A vital process and potential therapeutic target in chronic obstructive pulmonary disease. Inflammation Res: Off J Eur Histamine Res Soc. (2023) 72:1761–72. doi: 10.1007/s00011-023-01786-0 37695356

[63] Ma X Bi E Lu Y Su P Huang C Liu L. Cholesterol induces Cd8(+) T cell exhaustion in the tumor microenvironment. Cell Metab. (2019) 30:143–56.e5. doi: 10.1016/j.cmet.2019.04.002 31031094 PMC7061417

[64] Shuwen H Yinhang W Jing Z Qiang Y Yizhen J Quan Q. Cholesterol induction in Cd8(+) T cell exhaustion in colorectal cancer via the regulation of endoplasmic reticulum-mitochondria contact sites. Cancer Immunol Immunother: CII. (2023) 72:4441–56. doi: 10.1007/s00262-023-03555-8 PMC10991466 37919522

[65] Wang J Zhao X Wan YY. Intricacies of Tgf-B Signaling in Treg and Th17 cell biology. Cell Mol Immunol. (2023) 20:1002–22. doi: 10.1038/s41423-023-01036-7 PMC10468540 37217798

[66] Hou J Wang X Su C Ma W Zheng X Ge X. Reduced frequencies of Foxp3(+)Garp(+) regulatory T cells in copd patients are associated with multi-organ loss of tissue phenotype. Respir Res. (2022) 23:176. doi: 10.1186/s12931-022-02099-2 35780120 PMC9250745

[67] Jansen K Wirz OF van de Veen W Tan G Mirer D Sokolowska M. Loss of regulatory capacity in treg cells following rhinovirus infection. J Allergy Clin Immunol. (2021) 148:1016–29.e16. doi: 10.1016/j.jaci.2021.05.045 34153372

[68] Norlander AE Bloodworth MH Toki S Zhang J Zhou W Boyd K. Prostaglandin I2 signaling licenses treg suppressive function and prevents pathogenic reprogramming. J Clin Invest. (2021) 131:e140690. doi: 10.1172/jci140690 33529171 PMC8011897

[69] Ding Z Cai T Tang J Sun H Qi X Zhang Y. Setd2 supports Gata3(+)St2(+) thymic-derived treg cells and suppresses intestinal inflammation. Nat Commun. (2022) 13:7468. doi: 10.1038/s41467-022-35250-0 36463230 PMC9719510

[70] Christenson SA Steiling K van den Berge M Hijazi K Hiemstra PS Postma DS. Asthma-copd overlap. Clinical relevance of genomic signatures of type 2 inflammation in chronic obstructive pulmonary disease. Am J Respir Crit Care Med. (2015) 191:758–66. doi: 10.1164/rccm.201408-1458OC PMC4407484 25611785

[71] Becker EJ Faiz A van den Berge M Timens W Hiemstra PS Clark K. Bronchial gene expression signature associated with rate of subsequent Fev(1) decline in individuals with and at risk of copd. Thorax. (2022) 77:31–9. doi: 10.1136/thoraxjnl-2019-214476 PMC13021120 33972452

[72] Doyle AD Mukherjee M LeSuer WE Bittner TB Pasha SM Frere JJ. Eosinophil-derived Il-13 promotes emphysema. Eur Respir J. (2019) 53:1801291. doi: 10.1183/13993003.01291-2018 30728205 PMC7313423

[73] Shibata S Miyake K Tateishi T Yoshikawa S Yamanishi Y Miyazaki Y. Basophils trigger emphysema development in a murine model of copd through Il-4-mediated generation of Mmp-12-producing macrophages. Proc Natl Acad Sci U.S.A. (2018) 115:13057–62. doi: 10.1073/pnas.1813927115 PMC6305004 30510003

[74] Zheng T Zhu Z Wang Z Homer RJ Ma B Riese RJ Jr.. Inducible targeting of Il-13 to the adult lung causes matrix metalloproteinase- and cathepsin-dependent emphysema. J Clin Invest. (2000) 106:1081–93. doi: 10.1172/jci10458 PMC301418 11067861

[75] Cooper PR Poll CT Barnes PJ Sturton RG. Involvement of Il-13 in tobacco smoke-induced changes in the structure and function of rat intrapulmonary airways. Am J Respir Cell Mol Biol. (2010) 43:220–6. doi: 10.1165/rcmb.2009-0117OC 19783789

[76] Kolsum U Damera G Pham TH Southworth T Mason S Karur P. Pulmonary inflammation in patients with chronic obstructive pulmonary disease with higher blood eosinophil counts. J Allergy Clin Immunol. (2017) 140:1181–4.e7. doi: 10.1016/j.jaci.2017.04.027 28506852

[77] Romano Ibarra GS Lei L Yu W Thurman AL Gansemer ND Meyerholz DK. Il-13 induces loss of Cftr in ionocytes and reduces airway epithelial fluid absorption. J Clin Invest. (2024) 134:e181995. doi: 10.1172/jci181995 39255033 PMC11527443

[78] Contoli M Ito K Padovani A Poletti D Marku B Edwards MR. Th2 cytokines impair innate immune responses to rhinovirus in respiratory epithelial cells. Allergy. (2015) 70:910–20. doi: 10.1111/all.12627 25858686

[79] Laidlaw BJ Zhang N Marshall HD Staron MM Guan T Hu Y. Cd4+ T cell help guides formation of Cd103+ Lung-resident memory Cd8+ T cells during influenza viral infection. Immunity. (2014) 41:633–45. doi: 10.1016/j.immuni.2014.09.007 PMC4324721 25308332

[80] Lee S Yeung KK Watts TH. Tissue-resident memory T cells in protective immunity to influenza virus. Curr Opin Virol. (2024) 65:101397. doi: 10.1016/j.coviro.2024.101397 38458064

[81] Son YM Cheon IS Wu Y Li C Wang Z Gao X. Tissue-resident Cd4(+) T helper cells assist the development of protective respiratory B and Cd8(+) T cell memory responses. Sci Immunol. (2021) 6:eabb6852. doi: 10.1126/sciimmunol.abb6852 33419791 PMC8056937

[82] Reggio A Fuoco C Deodati R Palma A. Spp1 macrophages across diseases: A call for reclassification? FASEB J: Off Publ Fed Am Societies Exp Biol. (2025) 39:e70448. doi: 10.1096/fj.202403227R PMC11884386 40047497

[83] Wang C Li J Chen J Wang Z Zhu G Song L. Multi-omics analyses reveal biological and clinical insights in recurrent stage I non-small cell lung cancer. Nat Commun. (2025) 16:1477. doi: 10.1038/s41467-024-55068-2 39929832 PMC11811181

[84] Hoeft K Schaefer GJL Kim H Schumacher D Bleckwehl T Long Q. Platelet-instructed Spp1(+) macrophages drive myofibroblast activation in fibrosis in a Cxcl4-dependent manner. Cell Rep. (2023) 42:112131. doi: 10.1016/j.celrep.2023.112131 36807143 PMC9992450

[85] Singh R Belchamber KBR Fenwick PS Chana K Donaldson G Wedzicha JA. Defective monocyte-derived macrophage phagocytosis is associated with exacerbation frequency in copd. Respir Res. (2021) 22:113. doi: 10.1186/s12931-021-01718-8 33879129 PMC8059282

[86] Walton EL. Microbes are off the menu: defective macrophage phagocytosis in copd. Biomed J. (2017) 40:301–4. doi: 10.1016/j.bj.2017.12.002 PMC6138610 29433832

[87] Wang X Polverino F Rojas-Quintero J Zhang D Sánchez J Yambayev I. A disintegrin and a metalloproteinase-9 (Adam9): A novel proteinase culprit with multifarious contributions to copd. Am J Respir Crit Care Med. (2018) 198:1500–18. doi: 10.1164/rccm.201711-2300OC PMC6298633 29864380

[88] Xu X Yu T Dong L Glauben R Wu S Huang R. Eosinophils promote pulmonary matrix destruction and emphysema via cathepsin L. Signal Transduct Target Ther. (2023) 8:390. doi: 10.1038/s41392-023-01634-x 37816708 PMC10564720

[89] Doherty DF Nath S Poon J Foronjy RF Ohlmeyer M Dabo AJ. Protein phosphatase 2a reduces cigarette smoke-induced cathepsin S and loss of lung function. Am J Respir Crit Care Med. (2019) 200:51–62. doi: 10.1164/rccm.201808-1518OC 30641028 PMC6603057

[90] Christopoulou ME Papakonstantinou E Stolz D. Matrix metalloproteinases in chronic obstructive pulmonary disease. Int J Mol Sci. (2023) 24:3786. doi: 10.3390/ijms24043786 36835197 PMC9966421

[91] Ravi VM Neidert N Will P Joseph K Maier JP Kückelhaus J. T-cell dysfunction in the glioblastoma microenvironment is mediated by myeloid cells releasing interleukin-10. Nat Commun. (2022) 13:925. doi: 10.1038/s41467-022-28523-1 35177622 PMC8854421

[92] Mehta AK Kadel S Townsend MG Oliwa M Guerriero JL. Macrophage biology and mechanisms of immune suppression in breast cancer. Front Immunol. (2021) 12:643771. doi: 10.3389/fimmu.2021.643771 33968034 PMC8102870

[93] Zhang K Mishra A Jagannath C. New insight into arginine and tryptophan metabolism in macrophage activation during tuberculosis. Front Immunol. (2024) 15:1363938. doi: 10.3389/fimmu.2024.1363938 38605962 PMC11008464

[94] Stone TW Williams RO. Modulation of T cells by tryptophan metabolites in the kynurenine pathway. Trends Pharmacol Sci. (2023) 44:442–56. doi: 10.1016/j.tips.2023.04.006 37248103

[95] Yang X Deng B Zhao W Guo Y Wan Y Wu Z. Fabp5(+) lipid-loaded macrophages process tumour-derived unsaturated fatty acid signal to suppress T-cell antitumour immunity. J Hepatol. (2024) 82:676–89. doi: 10.1016/j.jhep.2024.09.029 39357545

[96] Wang J Li Y. Cd36 tango in cancer: signaling pathways and functions. Theranostics. (2019) 9:4893–908. doi: 10.7150/thno.36037 PMC6691380 31410189

[97] Huang SC Everts B Ivanova Y O’Sullivan D Nascimento M Smith AM. Cell-intrinsic lysosomal lipolysis is essential for alternative activation of macrophages. Nat Immunol. (2014) 15:846–55. doi: 10.1038/ni.2956 PMC4139419 25086775

[98] Yan J Horng T. Lipid metabolism in regulation of macrophage functions. Trends Cell Biol. (2020) 30:979–89. doi: 10.1016/j.tcb.2020.09.006 33036870

[99] Bidault G Virtue S Petkevicius K Jolin HE Dugourd A Guénantin AC. Srebp1-induced fatty acid synthesis depletes macrophages antioxidant defences to promote their alternative activation. Nat Metab. (2021) 3:1150–62. doi: 10.1038/s42255-021-00440-5 PMC7611716 34531575

[100] Fujii W Kapellos TS Baßler K Händler K Holsten L Knoll R. Alveolar macrophage transcriptomic profiling in copd shows major lipid metabolism changes. ERJ Open Res. (2021) 7:00915-2020. doi: 10.1183/23120541.00915-2020 34527724 PMC8435801

[101] Wu H Han Y Rodriguez Sillke Y Deng H Siddiqui S Treese C. Lipid droplet-dependent fatty acid metabolism controls the immune suppressive phenotype of tumor-associated macrophages. EMBO Mol Med. (2019) 11:e10698. doi: 10.15252/emmm.201910698 31602788 PMC6835560

[102] Qin R Ren W Ya G Wang B He J Ren S. Role of chemokines in the crosstalk between tumor and tumor-associated macrophages. Clin Exp Med. (2023) 23:1359–73. doi: 10.1007/s10238-022-00888-z PMC10460746 36173487

[103] Ma X Xie J Li B Shan H Jia Z Liu W. Weighted gene co-expression network analysis and single-cell sequence analysis uncover immune landscape and reveal hub genes of necroptosis in macrophages in myocardial ischaemia-reperfusion injury. Int Immunopharmacol. (2024) 140:112761. doi: 10.1016/j.intimp.2024.112761 39079349

[104] Xiao SM Xu R Yang YX Zhao R Xie Y Lei XD. Gastrointestinal stromal tumors regulate macrophage M2 polarization through the Mif/Cxcr4 axis to immune escape. Front Immunol. (2024) 15:1431535. doi: 10.3389/fimmu.2024.1431535 39464891 PMC11502962

[105] Ghoochani A Schwarz MA Yakubov E Engelhorn T Doerfler A Buchfelder M. Mif-Cd74 signaling impedes microglial M1 polarization and facilitates brain tumorigenesis. Oncogene. (2016) 35:6246–61. doi: 10.1038/onc.2016.160 27157615

[106] Wu L Wu W Zhang J Zhao Z Li L Zhu M. Natural coevolution of tumor and immunoenvironment in glioblastoma. Cancer Discov. (2022) 12:2820–37. doi: 10.1158/2159-8290.cd-22-0196 PMC9716251 36122307

[107] de Azevedo RA Shoshan E Whang S Markel G Jaiswal AR Liu A. Mif inhibition as a strategy for overcoming resistance to immune checkpoint blockade therapy in melanoma. Oncoimmunology. (2020) 9:1846915. doi: 10.1080/2162402x.2020.1846915 33344042 PMC7733907

[108] Figueiredo CR Azevedo RA Mousdell S Resende-Lara PT Ireland L Santos A. Blockade of Mif-Cd74 signalling on macrophages and dendritic cells restores the antitumour immune response against metastatic melanoma. Front Immunol. (2018) 9:1132. doi: 10.3389/fimmu.2018.01132 29875777 PMC5974174

[109] Song Z Wang X Liu X Luo Y Qiu J Yin A. Targeting of annexin A1 in tumor-associated macrophages as a therapeutic strategy for hepatocellular carcinoma. Biochem Pharmacol. (2023) 213:115612. doi: 10.1016/j.bcp.2023.115612 37209858

[110] Peng H Jiang L Yuan J Wu X Chen N Liu D. Single-cell characterization of differentiation trajectories and drug resistance features in gastric cancer with peritoneal metastasis. Clin Trans Med. (2024) 14:e70054. doi: 10.1002/ctm2.70054 PMC11488346 39422697

[111] Li Y Wang Z Lu F Miao Y Feng Q Zhu W. Novel T cell exhaustion gene signature to predict prognosis and immunotherapy response in thyroid carcinoma from integrated RNA-sequencing analysis. Sci Rep. (2024) 14:8375. doi: 10.1038/s41598-024-58419-7 38600248 PMC11006682

[112] Klement JD Paschall AV Redd PS Ibrahim ML Lu C Yang D. An osteopontin/Cd44 immune checkpoint controls Cd8+ T cell activation and tumor immune evasion. J Clin Invest. (2018) 128:5549–60. doi: 10.1172/jci123360 PMC6264631 30395540

[113] He H Chen S Fan Z Dong Y Wang Y Li S. Multi-dimensional single-cell characterization revealed suppressive immune microenvironment in Afp-positive hepatocellular carcinoma. Cell Discov. (2023) 9:60. doi: 10.1038/s41421-023-00563-x 37336873 PMC10279759

[114] Yang R Sun L Li CF Wang YH Yao J Li H. Galectin-9 interacts with Pd-1 and Tim-3 to regulate T cell death and is a target for cancer immunotherapy. Nat Commun. (2021) 12:832. doi: 10.1038/s41467-021-21099-2 33547304 PMC7864927

