# Supplementary material for: Developmental immune network of airway lymphocytes and innate immune cells in patients with stable COPD
Source: Front Immunol. 2025 Jun 16;16:1614655. doi: 10.3389/fimmu.2025.1614655 (PMC12206638; doi:10.3389/fimmu.2025.1614655)
Supplement: Supplementary file 1 [file DataSheet1.pdf]

frontiers

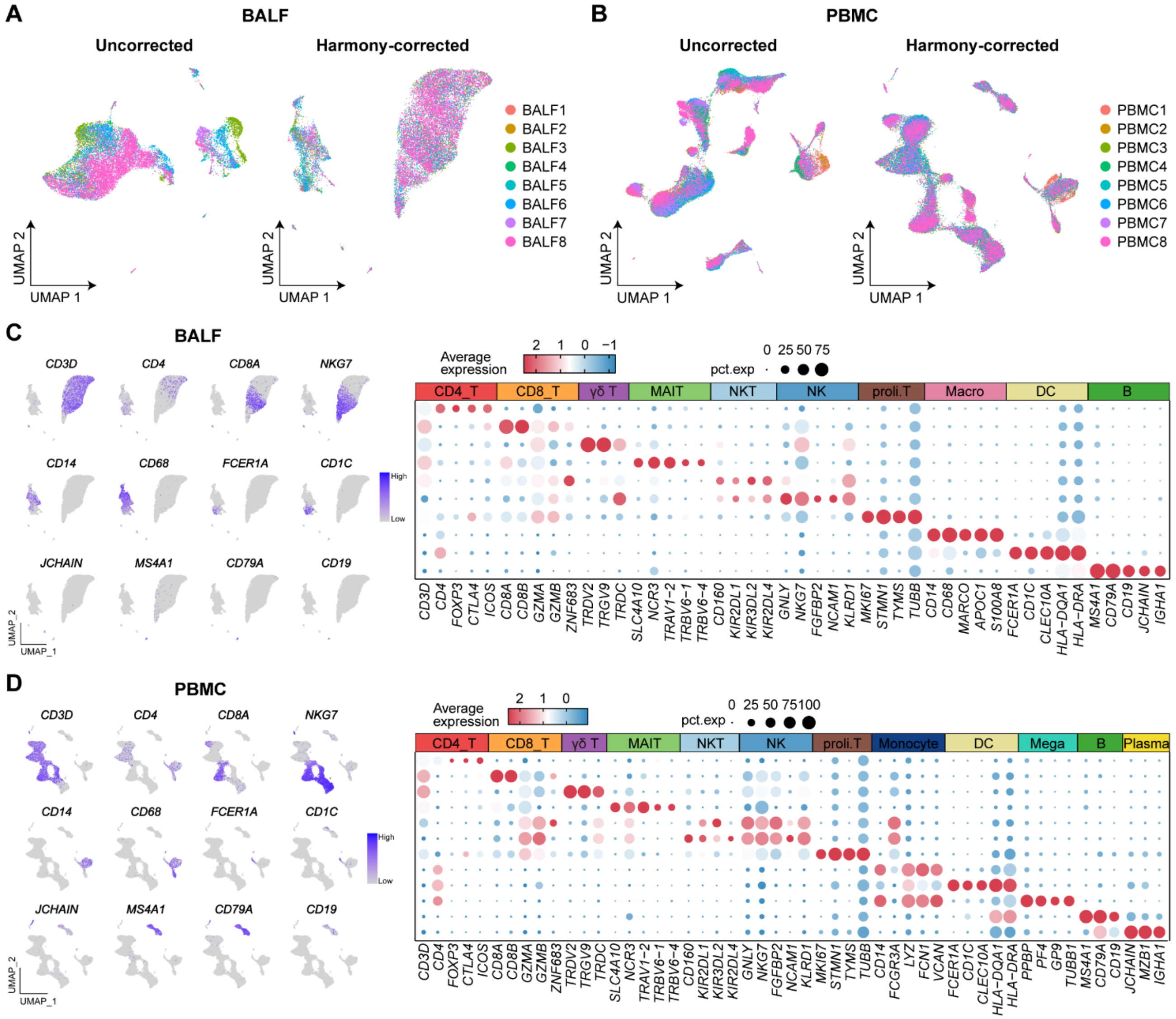

Supplementary Figure 1. Batch effect correction and immune cell characterization in BALF and PBMC. (**A**, **B**) UMAP plots showing cells before (left) and after (right) Harmony batch effect correction in BALF (A) and in PBMC (B). (**C**, **D**) Canonical marker genes of major immune cell types in BALF (C) and in PBMC (D).
