# Supplementary material for: Developmental immune network of airway lymphocytes and innate immune cells in patients with stable COPD
Source: Front Immunol. 2025 Jun 16;16:1614655. doi: 10.3389/fimmu.2025.1614655 (PMC12206638; doi:10.3389/fimmu.2025.1614655)
Supplement: Supplementary file 2 [file DataSheet2.pdf]

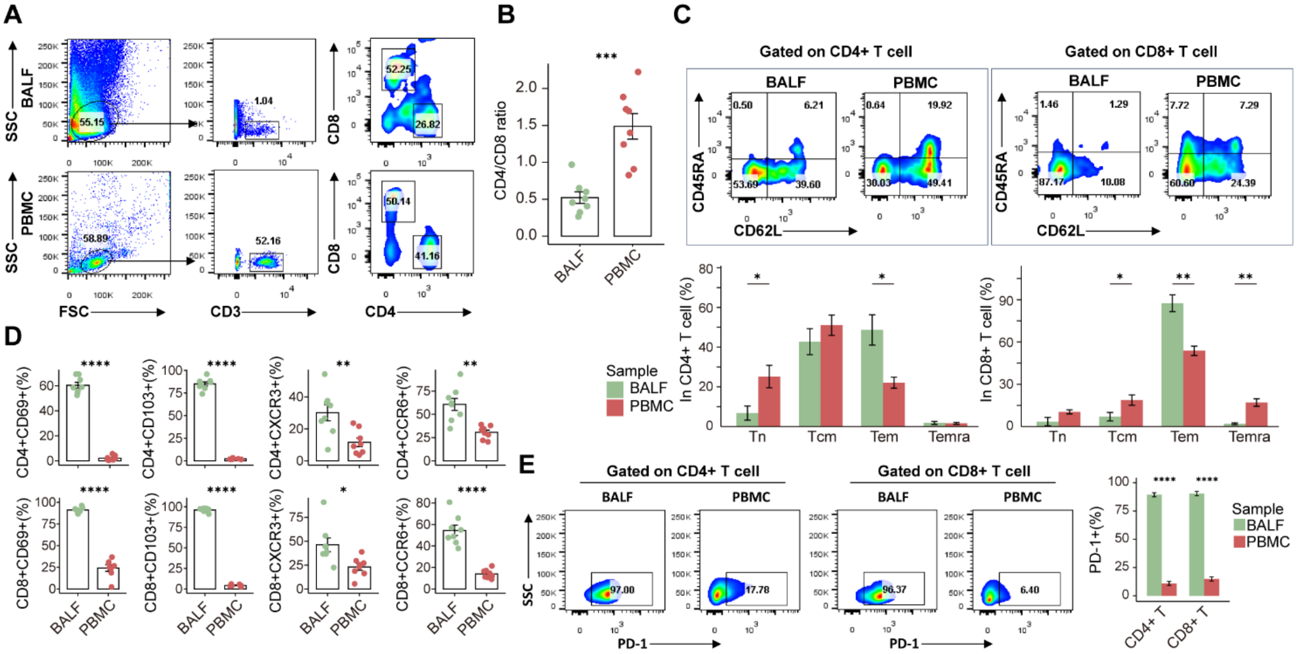

Supplementary Figure 2. Characteristics of CD4+ T and CD8+ T cells in BALF and PBMC. (**A**) Flow scatter plots showing the proportion of CD4+ T and CD8+ T cells in BALF and PBMC analyzed by flow cytometry. (**B**) Bar plot showing the ratio of CD4+ T cells to CD8+ T cells in BALF and PBMC analyzed by flow cytometry. (**C**) Flow scatter plots and bar plots showing the proportion of CD4+ T (left) and CD8+ T cell (right) subsets in BALF and PBMC. Tn, naive T cells; Tcm, central memory T cells; Tem, effector memory T cells; Temra, terminally differentiated effector memory CD45RA+ T cells. (**D**) Bar plots showing the expression of CD69, CD103, CXCR3, and CCR6 in CD4+ T and CD8+ T cells from BALF and PBMC analyzed by flow cytometry. (**E**) Flow scatter plots and bar plots showing the expression of PD-1 in CD4+ T and CD8+ T cells from BALF and PBMC. *$P \leqslant 0.05$, **$P < 0.01$, ***$P \leqslant 0.001$, and ****$P \leqslant 0.0001$ by Student's *t* test (B-E). BALF, n = 8; PBMC, n = 8.
