# Supplementary material for: Developmental immune network of airway lymphocytes and innate immune cells in patients with stable COPD
Source: Front Immunol. 2025 Jun 16;16:1614655. doi: 10.3389/fimmu.2025.1614655 (PMC12206638; doi:10.3389/fimmu.2025.1614655)
Supplement: Supplementary file 3 [file DataSheet3.pdf]

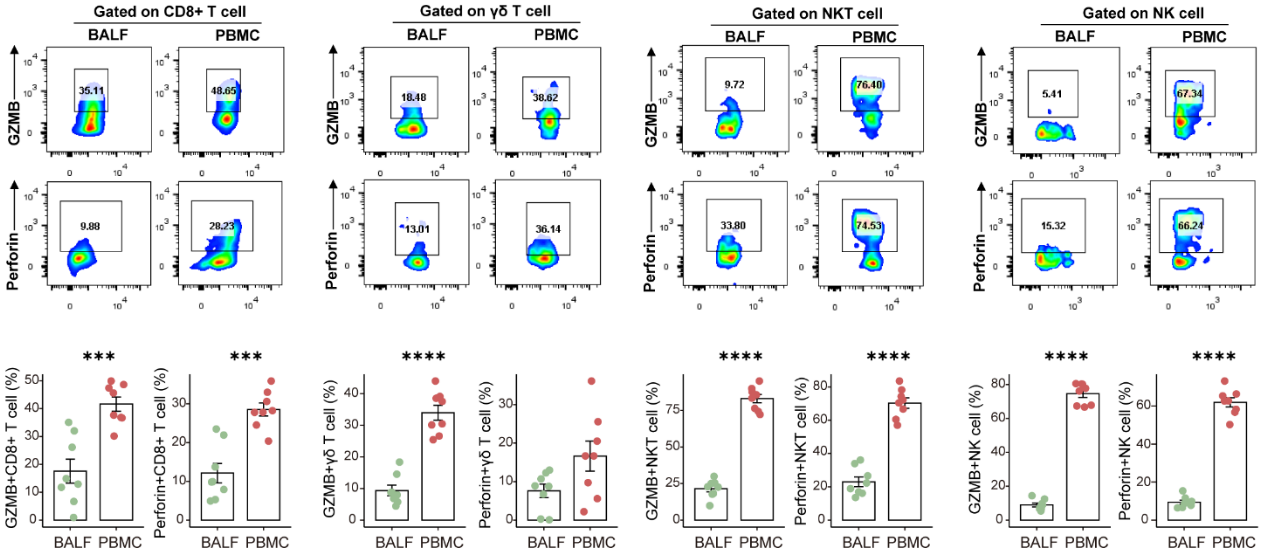

Supplementary Figure 3. Flow scatter plots and bar plots showing the expression of GZMB and perforin in CD8+ T, γδ T, NKT, and NK cells in BALF and PBMC. $***P \leqslant 0.001$ and $****P \leqslant 0.0001$ by Student's *t* test. BALF, n = 8; PBMC, n = 8.
