# Supplementary material for: Developmental immune network of airway lymphocytes and innate immune cells in patients with stable COPD
Source: Front Immunol. 2025 Jun 16;16:1614655. doi: 10.3389/fimmu.2025.1614655 (PMC12206638; doi:10.3389/fimmu.2025.1614655)
Supplement: Supplementary file 4 [file DataSheet4.pdf]

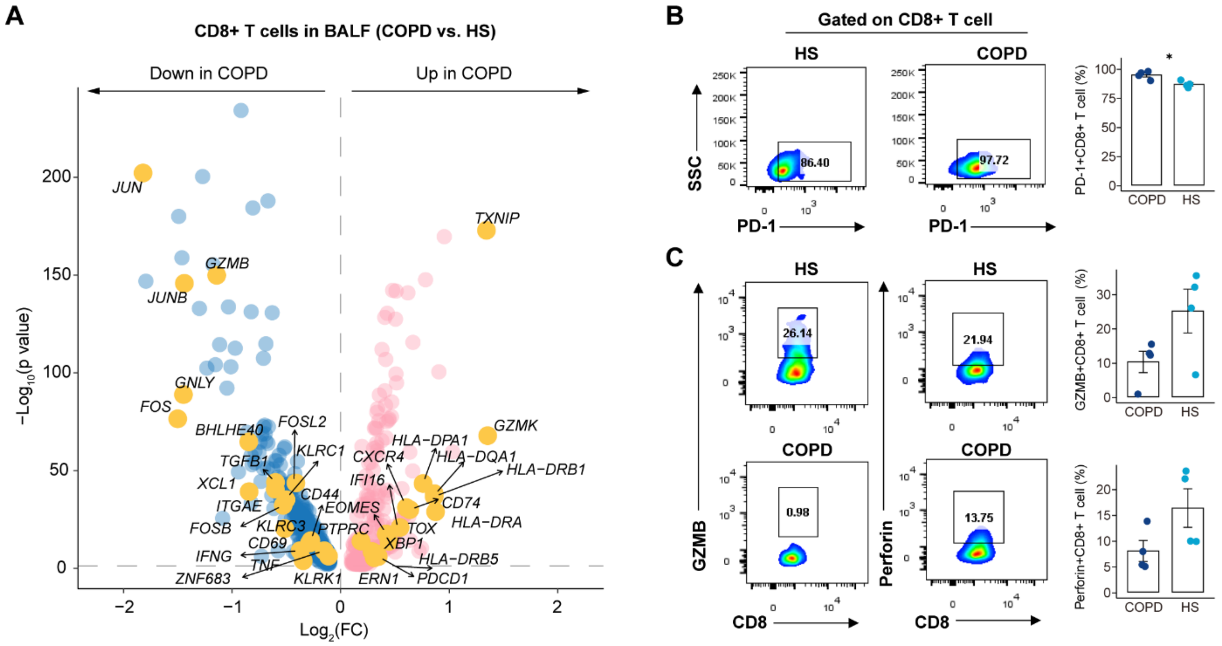

Supplementary Figure 4. Differences in CD8+ T cells between COPD and HS airways. (**A**) Volcano plot showing DEGs of CD8+ T cells in BALF between COPD and HS groups. (**B**, **C**) Flow scatter plots and bar plots showing the expression of PD-1 (B), GZMB, and perforin (C) in CD8+ T cells from HS and COPD airways. *$P < 0.05$ by Student's t test. HS, n = 4; COPD, n = 4.
