# Supplementary material for: Developmental immune network of airway lymphocytes and innate immune cells in patients with stable COPD
Source: Front Immunol. 2025 Jun 16;16:1614655. doi: 10.3389/fimmu.2025.1614655 (PMC12206638; doi:10.3389/fimmu.2025.1614655)
Supplement: Supplementary file 5 [file DataSheet5.pdf]

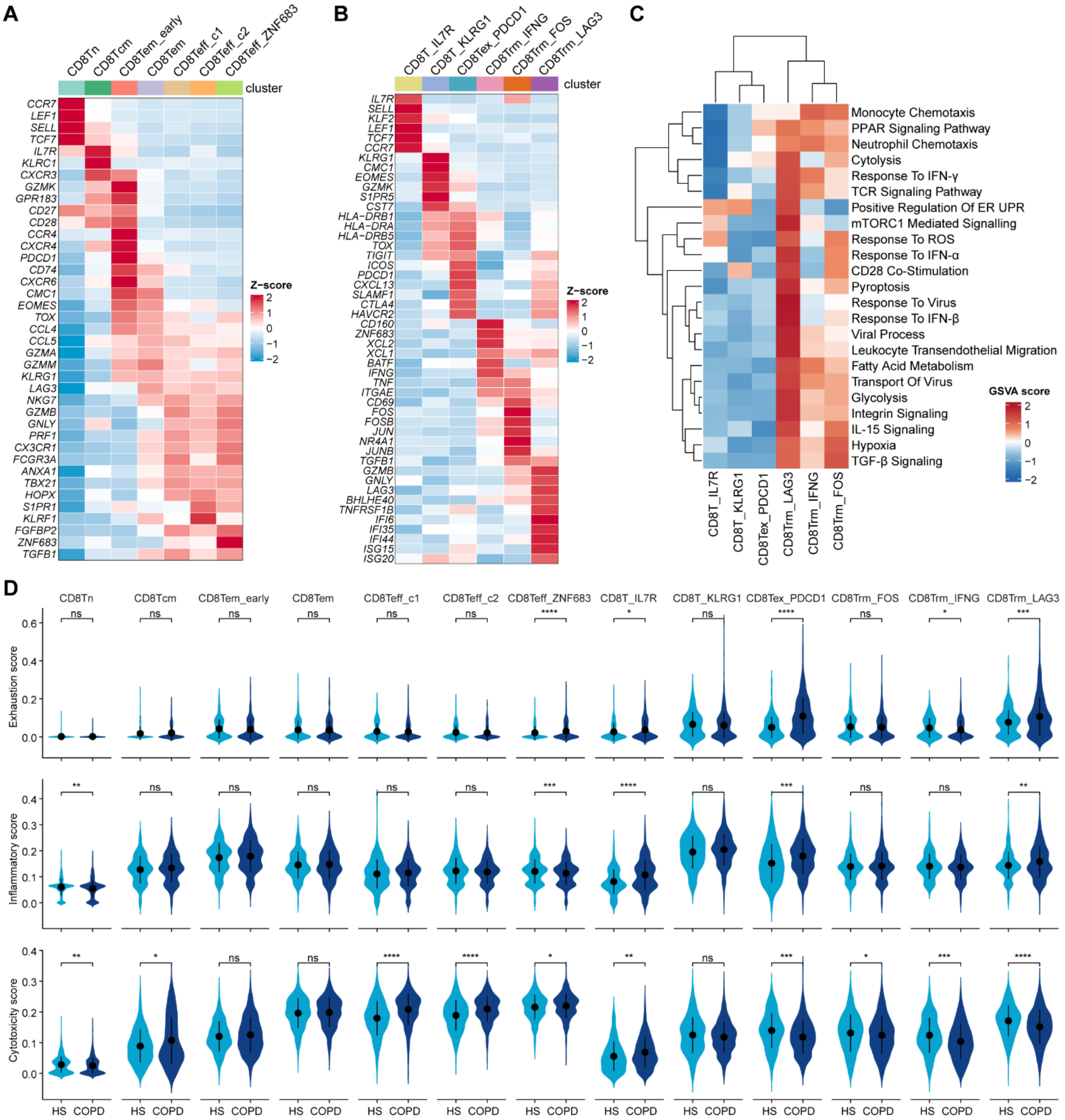

Supplementary Figure 5. Characteristics of CD8+ T cell subsets in BALF and PBMC. (**A**, **B**) Heatmap showing the signature genes of CD8+ T cell subsets in PBMC (A) and BALF (B). (**C**) Gene set variation analysis showing differentially activated pathways among the CD8+ T cell subsets in BALF. (**D**) Violin plots showing the signature gene set scores of CD8+ T cell subsets in HS and COPD groups. **P* < 0.05, ***P* < 0.01, ****P* < 0.001, and *****P* < 0.0001 by Wilcoxon test.
