# Supplementary material for: Developmental immune network of airway lymphocytes and innate immune cells in patients with stable COPD
Source: Front Immunol. 2025 Jun 16;16:1614655. doi: 10.3389/fimmu.2025.1614655 (PMC12206638; doi:10.3389/fimmu.2025.1614655)
Supplement: Supplementary file 6 [file DataSheet6.pdf]

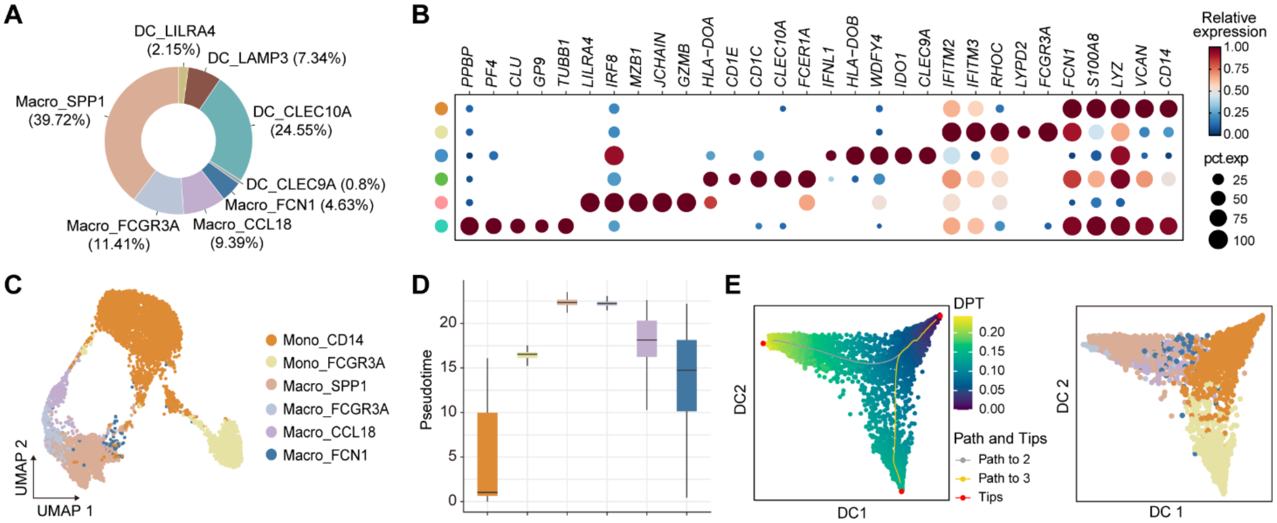

Supplementary Figure 6. Characteristics of myeloid cell subsets in BALF and PBMC. (**A**) Donut chart showing the proportion of myeloid cell subsets in BALF, colored by cell types. (**B**) Bubble heatmap showing the signature genes in myeloid cell subsets in PBMC. Dot color indicates the relative expression (scaled); dot size indicates the proportion of cells expressing the gene. (**C**) UMAP plot showing the integrated monocyte and macrophage subsets. (**D**) Box plots showing the pseudotime of monocyte and macrophage subsets, colored by cell types. (**E**) Diffusion maps showing the trajectories of blood monocytes and airway macrophages, colored by DPT (left) and cell types (right).
