# Supplementary material for: Developmental immune network of airway lymphocytes and innate immune cells in patients with stable COPD
Source: Front Immunol. 2025 Jun 16;16:1614655. doi: 10.3389/fimmu.2025.1614655 (PMC12206638; doi:10.3389/fimmu.2025.1614655)
Supplement: Supplementary file 7 [file DataSheet7.pdf]

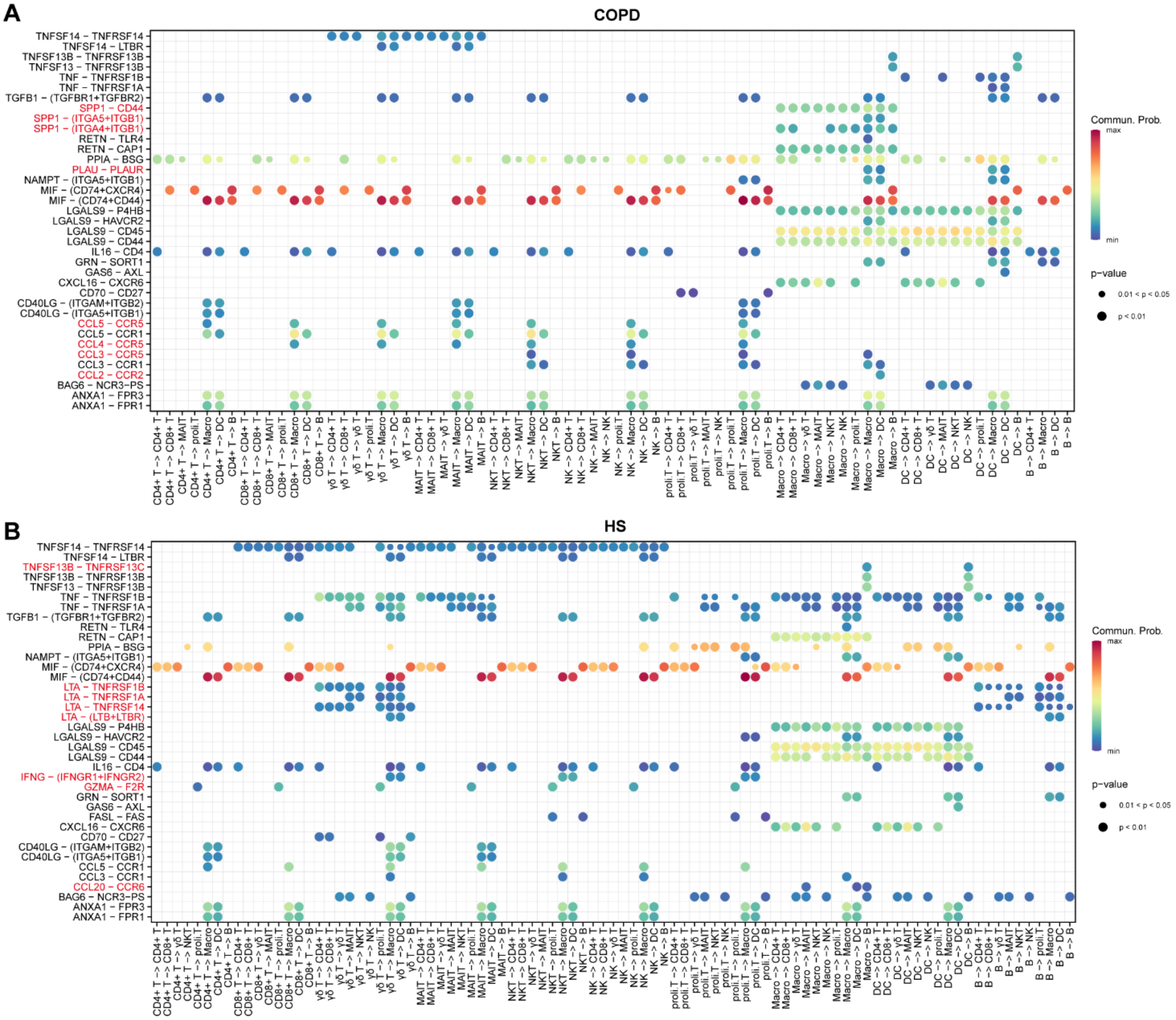

Supplementary Figure 7. The molecular interaction pairs of different immune cell type interaction pairs in COPD (**A**) and HS (**B**), and the molecule pair marker in red showing the COPD and HS specific molecular pairs.
